# Towards *in silico* Models of the Inflammatory Response in Bone Fracture Healing

**DOI:** 10.3389/fbioe.2021.703725

**Published:** 2021-09-30

**Authors:** Laura Lafuente-Gracia, Edoardo Borgiani, Gabriele Nasello, Liesbet Geris

**Affiliations:** ^1^ Biomechanics Section, Department of Mechanical Engineering, KU Leuven, Leuven, Belgium; ^2^ Prometheus: Division of Skeletal Tissue Engineering, KU Leuven, Leuven, Belgium; ^3^ Biomechanics Research Unit, GIGA *in silico* Medicine, University of Liège, Liège, Belgium; ^4^ Skeletal Biology and Engineering Research Center, KU Leuven, Leuven, Belgium

**Keywords:** bone regeneration, fracture healing, inflammatory response, *in silico* modeling, multiscale approach, experimental validation

## Abstract

*In silico* modeling is a powerful strategy to investigate the biological events occurring at tissue, cellular and subcellular level during bone fracture healing. However, most current models do not consider the impact of the inflammatory response on the later stages of bone repair. Indeed, as initiator of the healing process, this early phase can alter the regenerative outcome: if the inflammatory response is too strongly down- or upregulated, the fracture can result in a non-union. This review covers the fundamental information on fracture healing, *in silico* modeling and experimental validation. It starts with a description of the biology of fracture healing, paying particular attention to the inflammatory phase and its cellular and subcellular components. We then discuss the current state-of-the-art regarding *in silico* models of the immune response in different tissues as well as the bone regeneration process at the later stages of fracture healing. Combining the aforementioned biological and computational state-of-the-art, continuous, discrete and hybrid modeling technologies are discussed in light of their suitability to capture adequately the multiscale course of the inflammatory phase and its overall role in the healing outcome. Both in the establishment of models as in their validation step, experimental data is required. Hence, this review provides an overview of the different *in vitro* and *in vivo* set-ups that can be used to quantify cell- and tissue-scale properties and provide necessary input for model credibility assessment. In conclusion, this review aims to provide hands-on guidance for scientists interested in building *in silico* models as an additional tool to investigate the critical role of the inflammatory phase in bone regeneration.

## 1 Introduction

Bone healing is a complex, well-coordinated process that starts autonomously when a bone fracture occurs. Bone fractures are one of the most common injuries and their incidence in Europe is expected to increase by 23% over the coming decade due to ageing, as average life expectancy rises (Borgström et al., 2020). Owing to the bone tissue characteristics, successful healing is usually achieved within weeks (Marsh, 1998). However, up to 10% of bone fractures result in delayed healing or non-union (Zura et al., 2016). This risk rate is influenced by anatomical location, fracture severity and host factors such as age, smoking or the presence of comorbidities (Zura et al., 2016; Mills et al., 2017; Stewart, 2019). Current treatment options to prevent or cure these incidences present many drawbacks. Autologous bone grafting remains the gold standard procedure to treat non-unions, but this technique has limitations such as significant donor site morbidity and limited volume of available tissue (Pape et al., 2010). Alternative approaches to support the healing process, such as bone tissue engineering strategies, are still being tested in clinical trials or under development (Amini et al., 2012; Lammens et al., 2012; Papantoniou et al., 2021). These approaches mainly target the skeletal system and the repair phase of fracture healing, whereas recent findings have demonstrated that the skeletal and immune system are closely interacting through a carefully coordinated cross-talk between inflammatory and bone forming cells. Hence, inflammatory cells, such as macrophages, are believed to play a critical, but yet incompletely understood role in bone healing (Schlundt et al., 2018; Pajarinen et al., 2019).

In the last two decades, computational modeling has developed into a powerful technique to complement and reinforce traditional *in vitro* and *in vivo* experimentation, as it can provide an integrated view of the many events happening during the bone healing process and hence lead to a deeper understanding of said process. Moreover, computational models aim to reduce animal experimentation, although *in vivo* studies are often still required for validation purposes. Despite the importance of the inflammatory phase in bone fracture healing (Könnecke et al., 2014; Loi et al., 2016; Schmidt-Bleek et al., 2016), most computational models of bone regeneration focus on the repair phase, ignoring inflammation and its impact on the regenerative outcome. Therefore, modeling inflammation is a necessary inclusion in the current state-of-the-art, as it will allow to elucidate the mechanisms regulating the early phase of bone healing and their effect on the final regenerative outcome. This inclusion is not only needed, but it also starts now to be possible, as an increasing amount of experimental data regarding the inflammatory response is becoming available in the literature (Könnecke et al., 2014; Kovach et al., 2015; Schlundt et al., 2018; Wagar et al., 2018; Maruyama et al., 2020).

To date, despite the advances in the experimental work on the inflammatory phase of bone healing, no *in silico* models exist that capture the spatiotemporal dynamics of the process. In this review, we bring together the necessary components required to build a validated computational model able to predict the inflammatory response in bone healing and study the interaction of this phase with the subsequent phases of the healing process. First, Section 2 describes the overall bone fracture healing process with a strong focus on the inflammatory phase. Then, Section 3 revisits the current computational models describing the immune and the skeletal system responses after an injury. Next, Section 4 presents an overview of the experimental techniques that can be used throughout the development of computational models, from calibration to validation. Finally, the concurrence of biology, computational methods and experimental validation is discussed in Section 5. Taken together, this review aims to provide the necessary information and tools to build *in silico* models, which can provide an additional perspective to study the critical role of the inflammatory phase in bone regeneration.

## 2 The Biology of Bone Fracture Healing

Bones support the body, enable its mobility and protect vital organs. Moreover, bones produce hematopoietic cells and contribute to mineral storage within the bone marrow. Bone tissue is highly dynamic: bones adapt themselves to changes in the body, accommodating mechanical and biological requirements, and are constantly renewed in a process of remodeling. However, when stress and compression forces overcome bone tissue tolerance, bone fracture occurs (Oryan et al., 2015) and the process of fracture healing starts.

Bone fracture healing is described in Subsection 2.1. Subsection 2.2 focuses in more detail on the inflammatory response during fracture healing, paying special attention to cellular activity, cytokines and mechano-regulation.

### 2.1 Bone Fracture Healing Process

Bone can regenerate autonomously without fibrous scar formation after most cases of injury or fracture, eventually restoring its original state. This healing capacity is orchestrated by the complex fracture healing process, which involves multiple different cell types and is regulated by several biochemical, physical and mechanical factors (Einhorn, 1998). Depending on the mechanical stability of the fracture, direct or indirect healing will occur. Direct or primary fracture healing leads to restoration of the bone through a remodeling process. However, primary fracture healing is rather exceptional as it requires complete stability at the fracture site (Marsell and Einhorn, 2011), which is typically not achieved (Perren, 2002; Harwood et al., 2010; Claes et al., 2012). On the contrary, indirect or secondary fracture healing, the most common form of fracture healing (Marsell and Einhorn, 2011), is stimulated by interfragmentary motion (Harwood et al., 2010; Claes et al., 2012). In secondary fracture healing, bone repair advances *via* a multi-staged process involving both intramembranous and endochondral ossification (Loi et al., 2016), in which bone is formed directly from mesenchymal tissue or from intermediate cartilaginous tissue, respectively. However, high interfragmentary motion inhibits bone healing progression (Claes et al., 2012), resulting in compromised healing.

**FIGURE 1:**
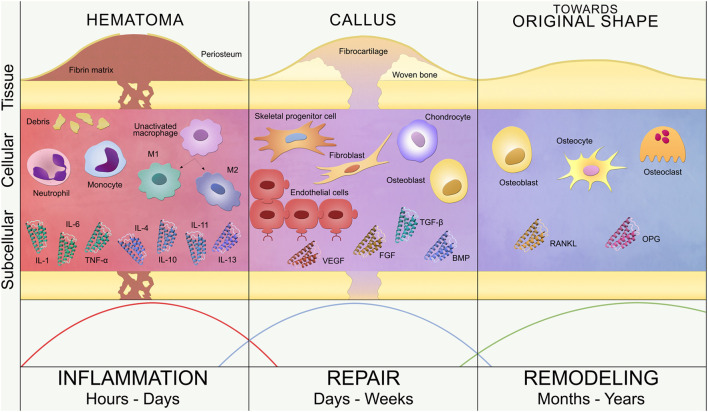
Bone fracture healing process. Timeline of secondary bone healing phases: inflammation, repair and remodeling. Tissue, cellular and subcellular levels are represented. Inflammation **(left):** hematoma formation triggers the invasion of inflammatory cells (neutrophils, monocytes and macrophages) and the release of pro-inflammatory (IL-1, IL-6, TNF-α) and anti-inflammatory (IL-4, IL-10, IL-11, IL-13) cytokines. Unactivated macrophages differentiate into classical (M1) and alternative (M2) activated macrophages. Repair **(center):** revascularization (endothelial cells), soft callus formation (fibrocartilage) and subsequent hard callus formation (woven bone) are regulated by repair cells (SPCs, fibroblasts, chondrocytes and osteoblasts) and growth factors (VEGF, FGF, BMP, TGF-β). Remodeling **(right):** restoration of the bone original shape by osteoblasts, osteocytes and osteoclasts, regulated by RANKL/OPG balance. These three phases are not rigidly defined over the timeline but overlap, as represented by the curves at the bottom of the image.

The classic phases of secondary fracture healing are inflammation, repair and remodeling (Figure 1). This simple classification is further elaborated in the contemporary literature, where additional overlapping substages have been proposed: hematoma formation, acute inflammation, granulation tissue formation, angiogenesis, fibrous tissue formation, fibrocartilage, soft callus development, cartilage mineralization, hard callus development, and, finally, remodeling (Kolar et al., 2010; Loi et al., 2016). Following Figure 1, these key events are briefly described below.

Immediately after the trauma, the fracture hematoma is formed due to the blood vessels disruption, which triggers the blood coagulation cascade, thus creating a fibrin network. This fibrin network serves as provisional extracellular matrix for the influx of inflammatory cells as well as the progenitor cells from the periosteum and the bone marrow (Kolar et al., 2010; Loi et al., 2016). Although this phase of bone healing is mostly defined as the invasion of inflammatory cells, the hematoma also contains immune cells present in the blood released from the disrupted vessels (Kolar et al., 2010). The healing process is then initiated with the activation of neutrophils, monocytes and macrophages (Kolar et al., 2010), leading to an acute inflammation reaction and the release of growth factors and cytokines. The initial fracture hematoma (Grundnes and Reikerås, 1993; Kolar et al., 2010) and subsequent inflammatory response (Bastian et al., 2011; Claes et al., 2012; Hoff et al., 2016) are critical for fracture healing (Schlundt et al., 2015). The hematoma is cleared in several days by the action of macrophages, which remove the fibrin matrix and necrotic cells at the bone ends *via* phagocytosis (Loi et al., 2016). A hypoxic environment remains within the fracture site, since neovasculature has not been developed yet.

At the end of the inflammatory phase, granulation tissue replaces the hematoma fibrin network due to the recruitment and proliferation of skeletal progenitor cells (SPCs) and fibroblasts (Harwood et al., 2010; Marsell and Einhorn, 2011). Granulation tissue favors angiogenesis, which is the formation of new blood vessels from pre-existing ones (Carano and Filvaroff, 2003). The vascularization process of the fracture site is promoted with interfragmentary motion during the early stages of fracture healing (Claes et al., 2012) and enhanced with angiogenic factors, such as fibroblast growth factor (FGF), platelet-derived growth factor (PDGF) or vascular endothelial growth factor (VEGF) (Barnes et al., 1999; Carano and Filvaroff, 2003; Tsiridis et al., 2007). Meanwhile, the hypoxic environment in the central region of the fracture site induces the differentiation of SPCs into chondrocytes (Tsiridis et al., 2007; Claes et al., 2012), starting the repair phase. Chondrocytes produce cartilage to connect the fractured bone ends, forming a soft callus that wraps the fracture gap. The soft callus provides initial mechanical stability and serves as scaffold for endochondral ossification during the repair phase (Harwood et al., 2010; Marsell and Einhorn, 2011; Loi et al., 2016). At the same time, SPCs differentiate into osteoblasts in the periosteal region away from the fracture site, hence creating woven bone *via* intramembranous ossification (Malizos and Papatheodorou, 2005; Claes et al., 2012; Loi et al., 2016). Both ossification events are regulated by growth factors, such as bone morphogenetic protein (BMP) and transforming growth factor beta (TGF-β), which control proliferation, differentiation and apoptosis of both chondrocytes and osteoblasts (Barnes et al., 1999; Tsiridis et al., 2007). As soft callus chondrocytes proliferate, they become hypertrophic and secrete VEGF, generating the rightful environment to attract blood vessels. Hypertrophic chondrocytes will finally undergo apoptosis and blood vessels will recruit progenitor cells that will differentiate into osteoblasts, leading to cartilage mineralization and generating the hard callus (Marsell and Einhorn, 2011). The formation of the hard callus entails the end of the repair phase of bone healing, leaving a solid and mechanically rigid fracture site, which has been revascularized and repopulated with bone cells. This stage is reached within several weeks or even months after the trauma (Loi et al., 2016) and generates the mechano-biological conditions to initiate the process of bone remodeling.

The remodeling phase is the final stage of the bone healing process and takes years to complete. Bone remodeling involves the resorption of immature woven bone and underlying cartilage matrix by osteoclasts, replacing these tissues with lamellar bone, as well as the decay of osteoblasts, undergoing apoptosis, or their maturation and embedding into the bone matrix as osteocytes. The cellular functions of osteoclasts and osteoblasts are regulated by cytokines such as receptor activator of nuclear factor kappa B ligand (RANKL) and osteoprotegerin (OPG). While RANKL promotes cell activation, differentiation and survival, OPG inhibits cell activation and induces apoptosis (Steeve et al., 2004; Tsiridis et al., 2007); leading to bone remodeling being controlled by the RANKL/OPG ratio (Steeve et al., 2004). The remodeling process establishes the osteon structure and Haversian system of the bone, restoring the bone’s original shape, strength and stability (Oryan et al., 2015; Loi et al., 2016).

### 2.2 Inflammatory Response to Bone Fracture

The inflammatory response is the immediate reaction to a trauma that starts when pathogenic agents enter the body due to a wound, generating undesired living conditions for the injured organism (Ward and Lentsch, 1999; Bastian et al., 2011; Loi et al., 2016). The inflammatory response consists of an automated cascade of signals that activates the innate immune system to contrast the invasion (Osuka et al., 2014). Cells of the innate immune system can directly attack the pathogen or trigger a second wave of signaling by releasing specific factors that can support the response (Stoecklein et al., 2012). Environmental conditions, such as swelling and temperature increase, are generated within the injured zone due to external attack encouraging a quicker inflammatory response (Evans et al., 2015; Loi et al., 2016).

The inflammatory response has a primary role on the overall bone healing process, and immune restricted patients are more prone to experience impaired healing (Hoff et al., 2017). For example, cytokines and growth factors released from macrophages recruit SPCs, promoting their capacity to colonize the fracture zone and differentiate, thus progressing the healing (Loi et al., 2016). Slower completion of bone fracture healing is observed in case of reduced influx of macrophages (Alexander et al., 2011; Schlundt et al., 2018). However, a continuously activated inflammatory response may incur a chronic state, which is also detrimental to successful healing (Osta et al., 2014). This chronic inflammatory fate was observed in numerous cases of delayed bone healing, where the prolonged exposition of the healing tissue to cytotoxic T cells extended the pro-inflammatory stage to the detriment of a fast and successful healing (Schmidt-Bleek et al., 2012). Adequate treatment of bone fracture healing should therefore generate a balanced response from the inflammatory stage. While it is known that the anti-inflammatory environment generates the conditions for a successful repair phase (Godwin et al., 2017), the prolonged use of nonsteroidal anti-inflammatory drugs was observed experimentally to alter the healing process (Lisowska et al., 2018). Inflammation involves a large number of agents that cooperate at different time and length scales to guarantee an adequate response. In the following subsections, we will describe the characteristics and functions of the principal immune cells and cytokines that are involved in this process.

#### 2.2.1 Cells of the Immune System

The cells involved in the inflammatory response can be divided into two groups according to their belonging to the innate or adaptive immune system (Medzhitov and Janeway, 1997). The cells of the innate immune system, which include monocytes, macrophages, neutrophils, natural killer cells and dendritic cells, constantly monitor the organism and provide the first response to the pathogens (Medzhitov and Janeway, 2000; Bouchery and Harris, 2019). The adaptive immune system guarantees the second pathogen-specific reaction and is mainly regulated by the migration of T and B lymphocytes, also referred to as T and B cells, within the infected region. This response is not immediate and requires more time to process and enter into action. However, the adaptive immune system can keep a copy of the antigen to accelerate the response in case of a future attack from the same pathogen. A full characterization of immune cells and their role in the inflammatory response is beyond the scope of the review and it is already well described elsewhere (Mosser and Edwards, 2008; Chaplin, 2010).

Cells of both the innate and adaptive immune system are present in the fracture site during the inflammatory stage of bone healing (Baht et al., 2018). For example, circulating neutrophils and monocytes migrate to the healing region in the first hours after the injury (Hoff et al., 2016; Kovtun et al., 2016). Neutrophils are the first cells to be recruited in the healing region to promote the formation of the fibrin thrombus to stabilize the fracture (Bastian et al., 2016). Monocytes circulate within the bloodstream, ready to extravasate from the capillaries to the surrounding tissues when the inflammatory response is triggered (Maslin et al., 2005). In the bone healing scenario, monocytes are also recruited from the bone marrow and they invade the fracture region to clean it from debris and to upregulate the pro-inflammatory response (Soltan et al., 2012). Once in the fracture site, monocytes will turn into adherent cells and differentiate into macrophages. The macrophages present within the fracture gap in the early stage of the inflammatory response are activated by the pro-inflammatory environment. Traditionally, macrophages were described to be activated into two states, named classically (M1) and alternatively (M2) activated, depending on whether they promote or inhibit the inflammatory response. Macrophage activation within the two states is fundamental for the right course of the inflammatory phase of bone healing. M1 macrophages regulate the initial pro-inflammatory response and clean up the region from dead cells and pathogenic agents through phagocytosis (Mescher, 2017). Additionally, they promote the recruitment of other pro-inflammatory cells through the secretion of specific cytokines (Gu et al., 2017). At the end of the inflammatory phase, the macrophages differentiate into M2 macrophages, which downregulate the inflammatory response to create the right environment for the following repair phase. The release of adequate anti-inflammatory cytokines provokes the recruitment of repair cells, such as SPCs and fibroblasts, which will start the rebuild of the fractured bone. The importance of the role of macrophages as initiators of the repair phase has been shown by Schlundt et al. (2018), who observed altered endochondral ossification in cases where the macrophages within the fracture site were depleted. Recently, experimental work has shown that macrophage activation is rather like a spectrum than a two-state system, with specific signatures depending on the location within the spectrum (Mosser and Edwards, 2008; Harasymowicz et al., 2021).

The adaptive response in fracture healing starts when T cells sense the molecular signals released from the cells of the innate immune system within the injury region. If the injury is characterized by infection from an external pathogen, specific antibodies are produced and released from B cells to accelerate the neutralization of the threat. The adaptive response in fracture healing follows a two-wave dynamics as it is observed to peak both after the fracture and later during the cartilage revascularization (Könnecke et al., 2014). Contrasting ideas are reported on the role of the adaptive immune response on the overall bone regeneration process: while fracture healing is observed to be accelerated when the adaptive reaction is suppressed (Toben et al., 2011), the positive effect of certain categories of T cells on bone regeneration has been also reported (Sadtler et al., 2016; Jahn and Weidinger, 2017). Furthermore, T and B cells promote the differentiation and recruitment of osteoclasts, which shift the balance of the later remodeling phase to favor bone resorption over formation (Manabe et al., 2001; Gillespie, 2007).

#### 2.2.2 Cytokines

Besides cells, the inflammatory response is mediated at subcellular level by molecular signals called cytokines. Cytokines are small proteins released from the inflammatory cells to regulate the inflammatory response, thus playing a role in the correct development of the early stages of bone healing. According to their ability to enhance or inhibit the inflammatory response, cytokines are typically divided into pro-inflammatory or anti-inflammatory, respectively.

Pro-inflammatory cytokines, such as interleukin 1 and 6 (IL-1, IL-6) and tumor necrosis factor alpha (TNF-α), are observed to peak their expression in the healing region within the first 24 h post-injury (Dimitriou et al., 2005). At subcellular level, TNF-α regulates the correct development of the inflammatory phase and, furthermore, it enhances the recruitment of SPCs to initiate the subsequent repair stage (Karnes et al., 2015).

Anti-inflammatory cytokines, which include different interleukins such as IL-4, IL-10, IL-11 and IL-13, are released to reduce inflammation when the first wave of pro-inflammatory response is over. Anti-inflammatory cytokines downregulate the inflammatory response and prevent chronic inflammation, which would be detrimental to fracture healing (Zhang and Yao, 2019).

#### 2.2.3 Mechano-Regulation

Although cells and cytokines are the major biological regulators of the inflammatory response in bone fracture healing, the micromovement in the interfragmentary region also regulates bone healing at cellular level. The early stage of bone healing is particularly sensitive to changes in mechano-stimulation, hence establishing an adequate mechanical environment at the injury site is necessary from the beginning of the inflammatory phase (Klein et al., 2003).

Monocytes, for example, express a stronger pro-inflammatory response under shear or compressive loading (Fahy et al., 2019). Mechano-regulation also affects the behavior of macrophages during the inflammatory phase as tissue stiffness influences their activation status, shape, mobility and phagocytic capacity (McWhorter et al., 2013; Adams et al., 2019; Jain et al., 2019; Gruber and Leifer, 2020). Elongation of macrophages under influence of mechanical loading induces anti-inflammatory activation and initiates the repair phase in the healing process (McWhorter et al., 2013). Thus, adequate fracture mechanical support is decisive to shape the macrophages and move from the inflammatory to the repair phase (Ballotta et al., 2014).

## 3 *In silico* Modeling

With the term *in silico*, scientists refer to the wide field of research that benefits from the use of computer modeling and simulation to investigate intricate and complex systems. This approach is becoming established in the biomedical field, as an additional resource to obtain a detailed understanding of the organism or its individual components. The flexibility provided by the computational approach favors the unveiling of aspects and insights that would be otherwise challenging to monitor experimentally. For this reason, the use of *in silico* clinical trials in all stages of the research and development pipeline has progressively gained more attention in the last decades (Viceconti et al., 2016). One of the more recent applications of *in silico* models is the execution of *in silico* clinical trials. In this context, the use of *in silico* models (e.g. through the use of Monte Carlo methods or the Bayesian approach) allows to quantify the parametric uncertainty in large data sets obtained from the results of the computational simulations. This is one way in which the effect of population variation can be captured *in silico*. To date, this approach is used by researchers to investigate the mechanisms behind neuron activation (Marder and Taylor, 2011), action potential stimuli in cardiac cells (Britton et al., 2013; Lawson et al., 2018), or mechanical properties of skeletal muscles (Sierra et al., 2015), among others.

**FIGURE 2:**
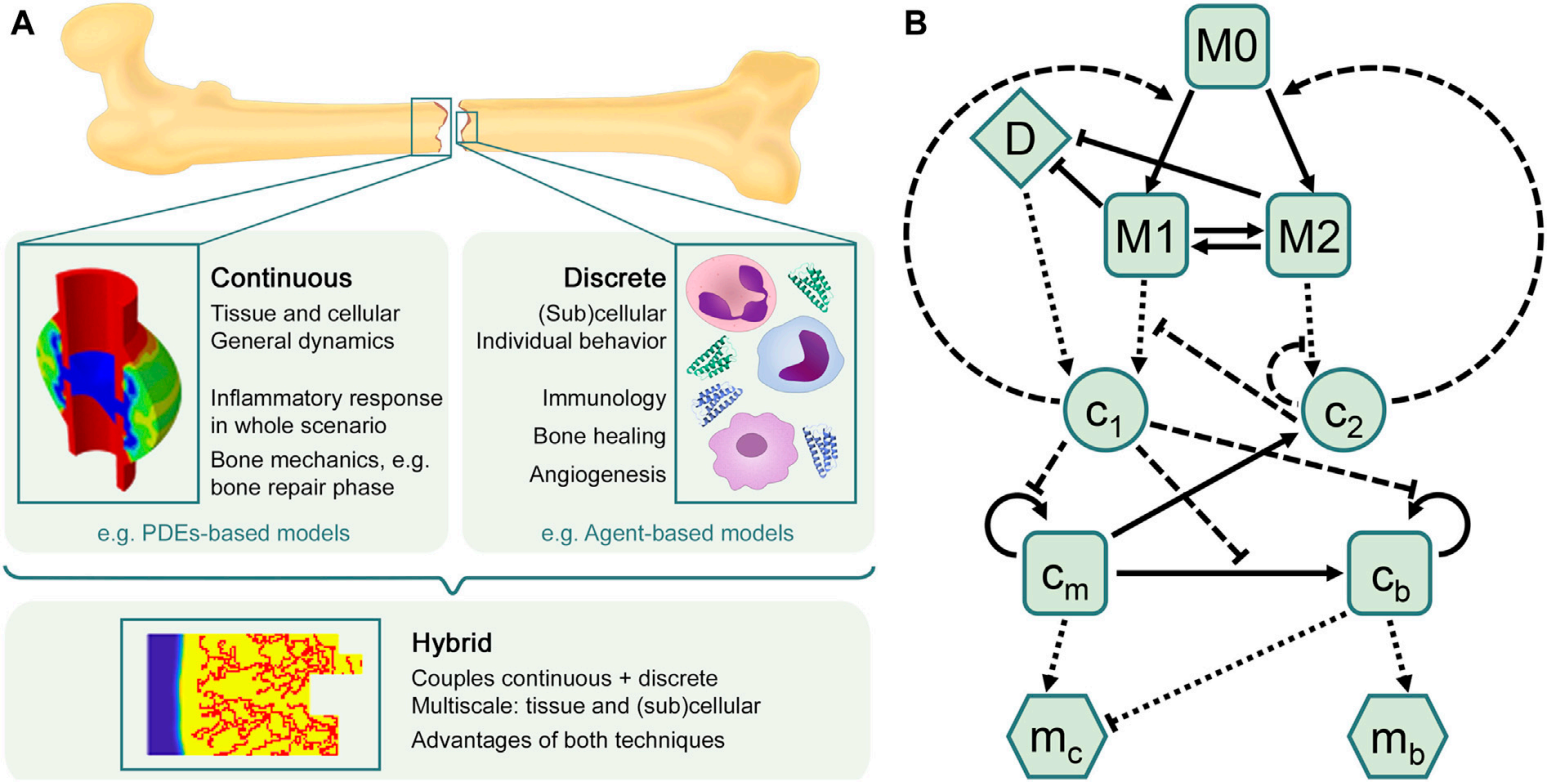
*In silico* approaches to model the bone healing process and the inflammatory response. **(A)** Overview of *in silico* techniques to describe biological processes and predict their different outcomes. The choice of the *in silico* model depends on the research goal. Continuous models are often used to describe general dynamics at tissue and cellular scales, such as bone mechanics, in which different tissue matrices interplay (figure adapted from Wang and Yang, 2018). Discrete models are mostly used to represent individual behavior at (sub)cellular scales, such as the immune response, which comprises a high number of cells and cytokines. The hybrid approach combines the advantages of both continuous and discrete techniques, providing comprehensive multiscale models that allow to investigate, for instance, sprouting angiogenesis during the bone regeneration process (figure obtained with the model described in Carlier et al., 2016). **(B)** Flow diagram summarizing the macrophage-mediated inflammation in bone fracture healing described in Trejo et al. (2019). Cells are represented by squares: unactivated macrophages (M_0_), classical macrophages (M_1_), alternative macrophages (M_2_), SPCs (c_*m*_) and osteoblasts (c_*b*_). Pro-inflammatory (c_1_) and anti-inflammatory (c_2_) cytokines are represented by circles. Tissue matrices are represented by hexagons: fibrocartilage (m_*c*_) and woven bone (m_*b*_). Debris (D) is represented by a diamond. Adapted from Trejo et al. (2019).

There is a wide range of *in silico* models available in the literature to investigate different aspects of bone regeneration *in silico* (see Doblaré et al., 2004; Giorgi et al., 2016; Borgiani et al., 2017; Ghiasi et al., 2017 for recent reviews), but most of them focus only on the repair and remodeling phases, thus ignoring the inflammation phase. At the same time, many approaches modeling immune and inflammatory responses in other tissues have been presented in the last two decades (see Section 3.2). The inflammatory response is already a complex process to simulate and, certainly, additional complexity arises when it is included in the bone healing model, as interactions and processes at different biological levels (tissue, cellular and subcellular) have to be considered. Besides, there are different computational approaches (continuous, discrete or hybrid) to model these different levels over their relevant time and length scales. The main characteristics of each approach are discussed in Subsection 3.1 and illustrated in Figure 2A, in order to elucidate which approach might be best to use depending on the biological goal of the research. Next, an overview is provided of the most relevant models to investigate the inflammatory response (Subsection 3.2) and the bone regeneration process (Subsection 3.3). In addition, Subsection 3.4 introduces the only *in silico* model that is, to the best of the authors’ knowledge, currently investigating the inflammatory response in bone fracture healing.

### 3.1 *In silico* Approaches to Model Biological Processes

There are different mathematical approaches to model a given biological situation: deterministic or stochastic, continuous or discrete over time and length scales, phenomenological or mechanistic. The appropriate approach is determined by the question that needs to be answered, the context of use, the available data and the computational resources. Even then, various models can produce qualitatively similar behavior (Anderson and Chaplain, 1998; Alber et al., 2006). Since the interest of an *in silico* model of the inflammatory response in bone fracture healing lies in understanding the biological events happening within the fracture region, we will focus here on models that incorporate physiological processes. To build such type of model, a common framework in mathematical biology is the so-called compartmental model.

A compartmental model is a system with different compartments and transitions between them (Figure 2B). In Figure 2B, specific biological entities (chemical factors, cells and extracellular matrices) have been assigned to one compartment depending on their type and can interact with entities in other compartments by transitions equipped with rates. As a result of interacting compartments, a coupled system of conservation equations is derived, in which each compartment is represented by one equation. Transitions between compartments represent biological processes such as migration, differentiation or apoptosis. Rates often follow the law of mass action and are modeled using rate formulations such as Michaelis-Menten kinetics or the Hill function. If the biological entities migrate either randomly or directed up to a gradient (such as chemotaxis or haptotaxis), a diffusion term is considered in the equation, providing a solution as function of time and space. Additionally, density-dependent models include growth dynamics by using e.g. a logistic-growth function (Murray, 1989). More details about common principles to model biological processes (in bone fracture healing) can be found in Table 1. Compartmental models can be translated into deterministic or stochastic models, using continuous- or discrete-time approaches. In most cases, the solution of the resulting system of equations is impossible to solve and represent using analytical techniques and hence it is approximated with numerical methods.

**TABLE 1:** Overview of the biological agents and processes in bone fracture healing and the way they can be captured *in silico* in continuous-time or discrete-time models. In keeping with the description in Section 3.1, the spatial scale (if present) is mentioned. Examples are provided of experimental setups to use during the calibration phase of *in silico* models. Units of quantitative parameters that can be extracted from experiments are in squared brackets. For those experiments that lead to qualitative observations, this is mentioned explicitly.

Type	Activity	Continuous-time models	Discrete-time models	Experimental techniques
Cells	Random motility	1) Fick’s second law, e.g. diffusion coefficient estimated from molecular weight or experimental data^a^; 2) Haptokinetic process, e.g. influenced by total matrix density such that cells cannot move in absence or abundance of ECM density^b^	Each agent moves in one of the empty surrounding positions chosen randomly by the algorithm^i,j,m–q^	Brightfield microscopy can quantify cell migration in organ-on-chip systems^r^ [cell velocity: distance/time]
	Chemotaxis	Receptor-ligand kinetics, e.g. maximum chemotactic response at certain growth factor concentration^c^	The selection of the surrounding position during migration is not random but it is biased by the concentration of the chemotactic factor^i,o,q^	Organ-on-chip systems facilitate the application of chemical gradients^r^ [diffusion of the leading edge of chemical: distance/time]
	Haptotaxis	Haptotactic process, e.g. based on a kinetic analysis of a model mechanism for the cell-surface-receptor-extracellular-ligand binding dynamics^b^	The selection of the surrounding position during migration is not random but it is biased according to the composition and fiber orientation of ECM^a,f,k^	Organ-on-chip systems facilitate the application of density gradients^r^ [binding concentration: mass/volume]
	Differentiation	Concentration-dependent curve, e.g. Hill function regulated by the concentration of growth factors^e^ or oxygen^f^ up to a saturation level	The agent changes its phenotype status according to the surrounding environmental conditions^j,n,q^	Analysis of cell surface markers (e.g. flow cytometry) [% of positive cells], gene expression profiles (e.g. qPCR and RNA-seq) [fold change in gene expression] and stainings (e.g. Alizarin Red for osteogenic differentiation)^s^ [qualitative observation]
	Polarization/activation	Concentration-dependent curve, e.g. Hill function regulated by the concentration of cytokines up to a saturation level^g^	The agent changes its activation status according to the surrounding environmental conditions^i,m,o,p^	Analysis of cell surface markers (e.g. flow cytometry) (% of positive cells) and gene expression profiles (e.g. qPCR and RNA-seq) (qualitative observation)^t^
	Proliferation	Fisher equation and logistic growth function such that rate of cell division decreases linearly with cell density, e.g. regulated by ECM density^b^ or oxygen tension^d^	A proliferative agent creates a copy of itself in one of the empty surrounding positions chosen randomly by the algorithm^j,l–q^	Proliferation assays based on DNA synthesis (e.g. EdU assay) [% of proliferating cells] or metabolic activity (e.g. MTT assay) (arbitrary units)^s^
	Apoptosis	1) Rate estimated from experimental data^e^; 2) Concentration-dependent curve, e.g. Hill function regulated by oxygen tension up to a saturation level^d^	The apoptotic agent is removed from the model^i,j,l,m,o,p,q^; nutrient-related survival conditions are applied by increasing the apoptosis ratio in undesired conditions^n^	Depending on the apoptosis stage, fluorimetric assays detecting mito-chondrial degradation, caspase activation or DNA fragmentation [% of viable cells]
	Senescence	Cell differentiation as evolutionary process, e.g. cells gain properties of another cell type gradually over time^g^	The senescent agent gradually reduces its cellular activity to zero (not performing actions, but not removed from the model)	Staining of SA-β-gal [qualitative observation]
Chemical agents (cytokines, growth factors, hormones, etc.)	Diffusion	Fick’s second law, e.g. diffusion coefficient estimated from molecular weight or experimental data^a^	Discretized Fick’s first law: the amount of substance exchanged between two adjacent patches is proportional to concentration difference, diffusing from patch of higher concentration to patch of lower one^i,p,q^	The biomolecule distribution across an hydrogel can be quantified with immunoassays (e.g ELISA) [biomolecule concentration: mass/volume]^r^
	Production	1) Rate estimated from experimental data^a^; 2) Concentration-dependent curve, e.g. Hill function to model a threshold-like behavior^e^	Substance concentration increases in function of the number of agents present in the patch according to a defined production ratio^i,n,p,q^	Immunoassays to quantify protein synthesis (e.g. ELISA)^u^ [biomolecule concentration: mass/volume]
	Consumption	Michaelis-Menten kinetic law, e.g. oxygen consumption by cells^h^	Substance concentration decreases in function of the number of agents present in the patch according to a defined consumption ratio^q^	Metabolites labeled with stable isotope tracers (e.g glucose consumption or fatty acid uptake) [normalized metabolite consumption: molarity/(time ⋅ mass)]^t^
	Denaturation	Rate estimated from experimental data^a^	Substance decay within patch decreases by following a time-dependent exponential function^i,o,p,q^	Biomolecule half-life estimation (e.g. pulse-chase analysis for cellular proteins) [time]
Extracellular matrix	Synthesis	Rate estimated from experimental data^b^	Matrix percentage increases within the patch where the cell is localized according to a synthesis ratio^j,q^	Cells/ECM growth can be evaluated with a Live-Dead viability/cytotoxicity staining [volume fraction: %]^v^
	Degradation	Rate estimated from experimental data^b^	Matrix percentage decreases within the patch where the cell is localized according to degradation ratio^q^	Level of biomolecules associated to degradation (e.g. hydroxyproline for collagen matrix) [biomolecule concentration: mass/volume]
Debris	Phagocytosis	Concentration-dependent curve, e.g. Hill function to model engulfing rate^g^	Phagocytic agent reduces the debris concentration within a defined radius of action^l,o^	Phagocytes culture (e.g. macrophages) with cellular debris or pathogens [cytokine concentration: mass/volume]^w^
Angiogenesis	Vessel formation	Migration (random and directed)^a^ and proliferation^c^ of endothelial cells, finally producing vascular matrix	Development of vasculature according to tip endothelial cell movement^a,f,k,n^	Microscopy imaging, brightfield^x^ or confocal^y^, of an endothelial cell monolayer during sprouting [sprout displacement: length

References: ^a^ Anderson and Chaplain (1998), ^b^ Olsen et al. (1997), ^c^ Geris et al. (2008), ^d^ Carlier et al. (2015), ^e^ Bailón-Plaza and Van Der Meulen (2001), ^f^ Carlier et al. (2012), ^g^ Trejo et al. (2019), ^h^ Prokharau et al. (2012), ^i^ Mi et al. (2007), ^j^ Checa et al. (2011), ^k^ Peiffer et al. (2011), ^l^ Martínez et al. (2012), ^m^ Pennisi et al. (2013), ^n^ OReilly et al. (2016), ^o^ Shi et al. (2016), ^p^ Gong et al. (2017), ^q^ Borgiani et al. (2021), ^r^ Moreno-Arotzena et al. (2014), ^s^ Groeneveldt et al. (2020), ^t^ Vats et al. (2006), ^u^ Zhang et al. (2017), ^v^ Guyot et al. (2014), ^w^ Fraser et al. (2009), ^x^ Del Amo et al. (2016), ^y^ Vaeyens et al. (2020), Abbreviations: SA*-*β*-*gal, senescence-associated β-galactosidase; ELISA, enzyme-linked immunosorbent assay; qPCR, quantitative polymerase chain reaction; ECM, extracellular matrix; RNA-seq, RNA-sequencing.

Continuous-time models use differential equations to describe mechano-biological processes. Differential equation models, whether ordinary (ODE), delay (DDE), partial (PDE) or stochastic (SDE), imply a continuous overlap of generations (Murray, 1989), thus describing the chronological time of biological phenomena. ODEs often describe how spatial-average biological entities change over time, simulating e.g. inflammatory responses (Kumar et al., 2004; Reynolds et al., 2006; Vodovotz et al., 2006; Trejo et al., 2019) or the bone healing process (Trejo et al., 2019; Lo et al., 2020) at tissue and cellular levels, and individual intracellular dynamics (Warrender et al., 2006; Peiffer et al., 2011) at subcellular level. DDEs model biological processes that not only depend on the current time, but also on an earlier time; representing e.g. hematopoiesis regulation (Mackey and Glass, 1977; Faria and Oliveira, 2020) or inflammatory responses (Nagaraja et al., 2014). PDEs describe the spatiotemporal evolution of biological entities, using e.g. reaction-diffusion equations to model bone healing within the fracture area (Bailón-Plaza and Van Der Meulen, 2001; Lacroix and Prendergast, 2002; Gómez-Benito et al., 2005; Isaksson et al., 2006; Geris et al., 2008 among others, see section 3.3) or angiogenesis (Olsen et al., 1997; Anderson and Chaplain, 1998). SDEs introduce random parameters in the model and are used to investigate e.g. bone remodeling (Sun et al., 2012; Jerez et al., 2018). One main advantage of continuous-time models is that they have been studied exhaustively in the last centuries, leading to many well-known numerical methods to determine their solutions, such as the finite element (FE) method (Zienkiewicz et al., 1977, used e.g. in Zysset et al., 2013; Coquim et al., 2018), the finite difference method (Liszka and Orkisz, 1980, used e.g. in Nagatani et al., 2006) and the method of lines (Hundsdorfer and Verwer, 2003, used e.g. in Geris et al., 2008). Some main disadvantages are that they usually fail to capture heterogeneous behaviors (Van Dyke Parunak et al., 1998; Wilkinson, 2009) and that the incorporation of new biological aspects is often not trivial (Yates et al., 2001; Fachada et al., 2007).

Discrete-time models use difference equations to study small-scale biological processes at (sub)cellular levels. Difference equations do not consider overlap between successive generations as they are solved for each time increment, involving an inherent delay to register changes (Murray, 1989). Discrete models are characterized by a stochastic nature, allowing the introduction of probabilistic rules, such as Monte Carlo methods (Lux, 2018), to describe each biological entity with its own properties and not as part of a population (Alber et al., 2006). The most common discrete approaches in biomedicine are agent-based (AB) and cellular automata (CA) models. AB models simulate the behavior of agents that can evolve generation after generation by changing their spatial position and internal properties. AB models are typically used to investigate cellular dynamics in response to environmental conditions, finding many applications in immunology (Martínez et al., 2012; Shi et al., 2016; Pappalardo et al., 2020 among others, see section 3.2) and bone healing (Checa et al., 2011; OReilly et al., 2016; Borgiani et al., 2019 among others, see section 3.3). CA models are a subgroup of AB models, hence they are often referred to as AB. However, while AB models are more focused on the single agent behavior to explore its impact on the overall scenario, the CA method is based on nearest-neighbor interactions governed by phenomenological rules (Anderson and Chaplain, 1998), meaning that interactions in CA models regard only neighbor regions. CA models are typically used to describe angiogenesis (Bentley et al., 2008; Peiffer et al., 2011; Carlier et al., 2012). One advantage of discrete-time modeling is its capacity to model each single element as an individual entity, allowing heterogeneous behaviors (Van Dyke Parunak et al., 1998; Wilkinson, 2009). Some main disadvantages are that the number of unknown parameters is usually high, and these rely crucially on the biological parameters obtained from experimental data (Murray, 1989). This often entails a reduction of precision and accuracy, resulting in model simplifications and approximations (Shi et al., 2016).

Continuous and discrete models can complement each other in hybrid models. In hybrid models, continuous- and discrete-time approaches are coupled through input/output variables to provide multiscale models describing mechano-biological processes at tissue, cellular and/or subcellular levels. The most common hybrid formulation in biomedicine couples a PDE system of reaction-diffusion equations with AB or CA models (Stéphanou and Volpert, 2016). The PDE system is often used to capture biomechanical stimuli and tissue mechanical properties (Checa et al., 2011; Zahedmanesh and Lally, 2012; Virgilio et al., 2015; Ceresa et al., 2018), which regulate the spatial distribution of cells modeled with an AB model. CA models are often used to describe angiogenesis, and coupled to PDE systems describing the spatiotemporal evolution of cells, tissue matrices and chemical factors (Peiffer et al., 2011; Carlier et al., 2012). It is also common to use continuous formulations to regulate the subcellular behavior of individual cells within a discrete model. For instance, ODEs regulating the intracellular behavior of endothelial cells (Peiffer et al., 2011; Carlier et al., 2012) or PDEs determining the molecular environment of individual cells (Warrender et al., 2006). The reader is referred to Stéphanou and Volpert (2016) for a review of hybrid modeling in biology.

### 3.2 Modeling the Inflammatory Response

Computational models describing the immune response, also known as computational immunology, can be broadly classified into two groups: those describing generic inflammatory responses after infection or trauma, and those simulating immune responses in specific tissues. Most approaches of the former group are continuous, whereas the latter are often simulated using discrete or hybrid models. Starting with the models describing generic inflammatory responses, Kumar et al. (2004) proposed a three-equation ODE model to simulate a simplified acute inflammatory response, able to predict healthy and negative outcomes. This model describes the relationships between the pathogen, which instigates the innate immune response, and early and late pro-inflammatory mediators (Kumar et al., 2004). Reynolds et al. (2006) considered three subsystems for different biological situations (non-specific local immune response, resting phagocytes and activated phagocytes) and merged them into a four-equation ODE model describing the generic acute inflammatory response to a pathogen. A bifurcation analysis of the model identified when the outcome was compromised depending on the administration of anti-inflammatory mediators (Reynolds et al., 2006). In the same year, Vodovotz et al. (2006) introduced a more elaborate mathematical model to simulate a non-specific acute inflammatory response after trauma, infection or hemorrhagic shock. This ODE system described the dynamics of cells and cytokines and included the effect of tissue dysfunction, coagulation elements and blood pressure. In addition, it was the first model validated with animal and human experimental data (Vodovotz et al., 2006). Almost a decade later, Nagaraja et al. (2014) presented a comprehensive mathematical model to represent the local inflammation process in a wound and characterize the indicators triggering chronic inflammation. This model was validated with experimental data and consisted of fifteen ODEs and one DDE: the ODEs described inflammatory cells, cytokines and growth factors, whereas the DDE represented monocyte differentiation into pro-inflammatory macrophages, driven by chemotaxis with a 12 h delay (Nagaraja et al., 2014).

Within *in silico* models of tissue-specific immune responses, the popularity of AB methods is clear (Fachada et al., 2007; Shi et al., 2016). Several AB models of the immune system can be found in the literature, together with a large variety of simulators to develop them. Some models are implemented in custom AB simulators, such as ImmSim (Celada and Seiden, 1992; Seiden and Celada, 1992) and UISS (Pappalardo et al., 2010), whereas other models use generic open-source simulators that allow for the implementation of AB models such as NetLogo (Mi et al., 2007; Brown et al., 2011; Pennisi et al., 2013; Shi et al., 2016). ImmSim was the first AB model and framework to simulate the immune system, focusing on the processing of antigens and their effects on the different cell types (Celada and Seiden, 1992; Seiden and Celada, 1992; Fachada et al., 2007). Mi et al. (2007) presented an AB model focused on the interrelation between inflammation and skin wound healing in a physical domain. Skin injury and the subsequent inflammatory response were simulated to examine the general healing progression in terms of cells and cytokines dynamics. Brown et al. (2011) described a model of inflammation simulating the response of macrophages and fibroblasts to particulate exposure in the lung, as well as their interactions within the simulated environment, such that cytokines production, tissue damage and collagen deposition are represented. Martínez et al. (2012) developed a model of macrophage action on endocrine pancreas, focused on modeling the activation of the innate immune system upon stimulation by necrotic or apoptotic cell death in the first step of type 1 diabetes autoimmune response. Wendelsdorf et al. (2012) designed the ENISI simulator to represent mucosal inflammatory and regulatory immune pathways in the gut. Shi et al. (2016) proposed an integrated-mathematical-AB model to simulate the hepatic inflammatory response to *Salmonella* infection in mouse, which might cause a severe immune response and result in sepsis. Pennisi et al. (2013) and Pappalardo et al. (2020) investigated the cause of chronic inflammation in relapsing remitting multiple sclerosis using AB techniques. This framework was further developed into the Universal Immune System Simulator (UISS), which is now also used to investigate immunotherapy in cancer (Gianì et al., 2018) and the development of vaccines for Tuberculosis (Russo et al., 2020b) and Sars-Cov2 (Russo et al., 2020a).

Warrender et al. (2006) described a hybrid model of early *Mycobacterium* infection, the causative agent of tuberculosis, and the subsequent inflammatory response using a simulator called CyCells. With this approach, cells are represented explicitly and extracellular molecular components are represented by their concentration. More recently, Ceresa et al. (2018) presented a multiscale model coupling FE and AB techniques to simulate the immunological and biomechanical implications of emphysema, one of the major obstructive lung diseases. This model provided a detailed description of inflammation and tissue remodeling, since the AB part was based on existing ODE models of inflammation and immunological response and the FE part captured the biomechanical effects of repeated strain on the biological tissue (Ceresa et al., 2018).

### 3.3 Modeling the Repair and Remodeling Phases in Bone Healing

The use of computer models to simulate bone healing can be dated back to Carter et al. (1988), who investigated *in silico* the role of intermittent stress on the revascularization and tissue differentiation processes in the initial stages of bone healing. In the following years, many other studies exploited the computational power to study the mechano-regulation of the bone healing process (Prendergast et al., 1997; Carter and Beaupré, 1998; Claes and Heigele, 1999; Bailón-Plaza and Van Der Meulen, 2003; Isaksson et al., 2006; Geris et al., 2010; Checa et al., 2011; Burke and Kelly, 2012; Vetter et al., 2012; Borgiani et al., 2015; Wang and Yang, 2018). Another common application of computational methods is the simulation of the revascularization process on bone healing to highlight the role of angiogenesis and relative oxygen supply on disrupted tissues (Geris et al., 2008; Peiffer et al., 2011; Carlier et al., 2012; Carlier et al., 2015; OReilly et al., 2016). Moreover, different *in silico* models have been developed to investigate critical healing therapeutic strategies, such as the use of bone graft with a scaffold support (Perier-Metz et al., 2020), the transplant of stem cells (Geris et al., 2010; Carlier et al., 2016) or the provision of exogenous growth factors (Moore et al., 2014; Ribeiro et al., 2015) within the healing region. Different biomechanical studies employed *in silico* approaches to evaluate the impact of fracture stabilization (Gómez-Benito et al., 2006), gap size (Gómez-Benito et al., 2005) and nature of mechanical stimuli (Epari et al., 2006; García-Aznar et al., 2007; Steiner et al., 2013).

Most of the aforementioned studies use FE analyses to reproduce the mechanical environment (e.g. stress/strain distribution, tissue mechanical properties, bone density) within the injury. However, to date, many studies in this field started to additionally employ AB models to acquire a different point of view on the investigation of the mechano-biological relationships driving bone fracture healing. The supporting AB models are commonly employed to simulate the dynamics of repair cells (Byrne et al., 2011; Checa et al., 2011; Borgiani et al., 2019, 2021) and angiogenesis (Peiffer et al., 2011; Carlier et al., 2012; OReilly et al., 2016). Carlier et al. (2012) developed the hybrid MOSAIC model to simulate sprouting angiogenesis in a discrete environment. The behavior of the discrete endothelial cells was regulated by their protein levels and their relationship with cells, tissue and growth factors present in the global continuous environment. The multiscale model from Checa et al. (2011) investigated the inter-species differences in bone fracture healing between small and large animals within a mechano-regulated environment. They used an AB model to simulate how the spatial distribution of specialized bone repair cells (microenvironment) is regulated according to the mechanical stimulus predicted with FE (macroenvironment). Multiscale *in silico* modeling is a successful approach to explore the bone healing process at the levels of tissues, cells and subcellular agents by simulating their response to mechano-biological stimuli.

### 3.4 First Model of the Inflammatory Response in Bone Healing

The *in silico* studies of the repair and remodeling phases reported in the previous section do not include the simulation of the early stages of bone fracture healing, thus ignoring the role of the inflammatory response. Inflammation is characterized by numerous actors whose role in the overall scenario is worthy to be investigated. However, due to its complex nature, the inflammatory response to bone injury has been rarely simulated with a computer model. To the best of the authors’ knowledge, only one *in silico* model describing the inflammatory response in bone fracture healing has been reported in the literature. The model was first introduced by Kojouharov et al. (2017) and further updated within the same research group by Trejo et al. (2019).

Kojouharov et al. (2017) developed an eight-equation ODE model to simulate the temporal dynamics of debris, cells, cytokines and tissues from the first hours post-fracture, capturing the interaction between biological elements acting at multiple levels. Debris removal was modeled with a constant rate depending on the debris and macrophage densities, while the macrophages density depended on migration and emigration rates. The concentration of pro- and anti-inflammatory cytokines was simulated using Hill functions to capture a saturation effect, which depended on the concentration of debris and macrophages and of SPCs, respectively. Finally, the dynamics of SPCs, osteoblasts, fibrocartilage and woven bone was described as in Bailón-Plaza and Van Der Meulen (2001). The model simulated the biological time-dynamics in different case scenarios, highlighting the influence of a controlled cytokine concentration level as treatment to obtain an overall successful healing. Moreover, the model was employed to propose cytokine-based treatment in challenging healing conditions. For example, the model showed faster acceleration when an optimized dose of anti-inflammatory cytokines was administered at the beginning of the healing process.

Two years later, Trejo et al. (2019) incorporated two additional equations to simulate the distinction between classical and alternatively activated macrophages, and the ODE system was adapted accordingly (Figure 2B). Other biological processes were upgraded as well, the most relevant being debris removal, modeled now by a Hill function to represent the saturation of phagocytosis by macrophages, and macrophages migration, described now by a logistic growth function. The updated model allowed to analyze the role of macrophage activation status in the inflammatory phase to generate a successful signaling cascade initiating the subsequent repair phase. The model endorsed macrophages as promoters of tissue production during healing, giving further merit of this enhancement to the alternatively activated ones (M2). However, no spatial distribution of the different biological agents was modeled, as only temporal evolutions were reported as results.

The spatiotemporal evolution is of upmost importance to further explore the dynamics of all the involved actors during the progress of the inflammatory response, as it represents the heterogeneous distributions within the region of interest. For example, one hypothesis would be that macrophage migration to the fracture zone will initially have a bigger impact in the peripheral area and less effect in the central area, generating different spatial dynamics in the healing process. Therefore, we believe that the next generation of *in silico* fracture healing models should include both temporal and spatial evolution of the densities and concentrations of the different biological agents related to the inflammation phase. Moreover, many experimental studies investigate the immune response nowadays, as presented in Section 4. The incorporation of the spatial description *in silico* would allow a stronger validation of the future computational models investigating bone fracture healing from the initial inflammatory response to the later remodeling phase.

## 4 Experimental Validation of *in Silico* Models

**FIGURE 3:**
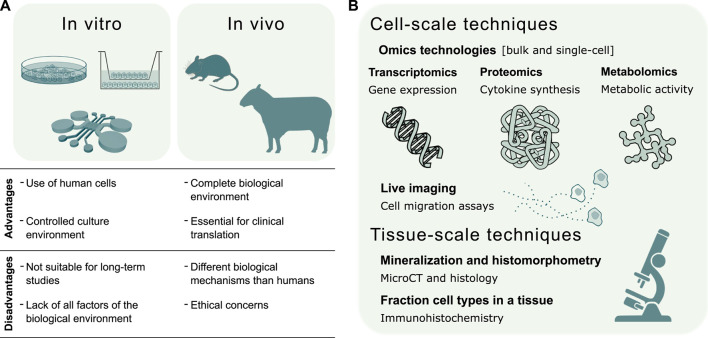
Validation of *in silico* models of the inflammatory response in bone healing. **(A)** Summary of *in vitro* and *in vivo* experimental techniques to validate the predictive capacity of *in silico* models. The choice of the experimental model depends whether the validation regards a specific mechanism or the global response. *In vitro* models investigate single biological mechanisms, such as the chemoattractant effect of inflammatory markers or specific cell types. *In vivo* models evaluate the effects of individual factors, such as the depletion of a cell type, on the complete biological response. **(B)** Experimental techniques for the validation of *in silico* models can be broadly divided into cell and tissue-scale techniques. The former validate *in silico* models of molecular mechanisms regulating cell function and models of cell migration dynamics. The latter validate *in silico* models of the repair and remodeling phase, by quantifying bone histomorphometric parameters, and models describing cellular composition in the fracture site.

Experimental techniques are continuously evolving to study the inflammatory response on multiple scales, ranging from micro-scale *in vitro* systems to large *in vivo* animal models. These results provide important information also in view of validating the predictive capacity of bone healing *in silico* models. Each modeling technique has its unique advantages and provides essential information about the inflammatory process (Figure 3A). *In vitro* models allow the culture of human cells in a controlled environment outside of living organisms, although they are poorly suited for long-term studies. Moreover, *in vitro* models may fail in recapitulating a clinically relevant environment due to the absence of all factors present *in vivo* (Boussommier-Calleja et al., 2016), which motivates the use of animal models. The resemblance of the human biological environment is the reason why *in vivo* models are an absolute requirement for translational studies of human immunology (Wagar et al., 2018). However, biological mechanisms may differ between animal models and humans (Mestas and Hughes, 2004).

Hereafter, both traditional and advanced *in vitro* and *in vivo* systems to model the inflammatory response in bone healing are discussed in Subsection 4.1 and Subsection 4.2, respectively. In Subsection 4.3 we describe different assays to extract both qualitative and quantitative data for the validation of *in silico* predictive models.

### 4.1 *In vitro* Models of the Inflammatory Response in Bone Healing

#### 4.1.1 Source of Inflammatory Cells

Human blood is the most frequently used source of immune cells for *in vitro* experiments since peripheral blood samples are easy to obtain. Immune cells with a single nucleus can be isolated from the whole peripheral blood by density centrifugation (Dagur and McCoy, 2015). These cells, named peripheral blood mononuclear cells (PBMCs), are a heterogeneous cell population mainly composed of lymphocytes and monocytes. Lymphoid cells account for 85% of all human PBMCs and consist of T cells (∼60%), B cells (∼10%) and natural killer (NK) cells (∼10%). Monocytes constitute around 15% of the total PBMCs count, while other cell types, such as dendritic cells, are less than 1% (Bittersohl and Steimer, 2016).

In general, *in vitro* experiments study specific cellular functions and require the isolation of single cell types. While monocytes are traditionally isolated from the rest of PBMCs and differentiated into macrophages by cell adhesion to tissue culture plastic (Rios et al., 2017), every cell type in PBMCs can be separated by labeling with magnetic beads. Immunomagnetic cell separation consists of binding magnetic beads to cell surface antigens using specific antibodies (Plouffe et al., 2015). The characteristic surface molecules, named cluster of differentiation (CD) molecules, of each PBMC type are known: CD3^+^ for T cells, CD22^+^ for B cells, CD56^+^/CD16^+^ for NK cells and CD14^+^ for monocytes (Bittersohl and Steimer, 2016).

Although human PBMCs are routinely used to answer fundamental questions about the immune cell functions, their choice has some drawbacks. First, immunomagnetic cell separation requires expensive reagents, such as antibodies. Secondly, *in vitro* results obtained from primary human cells are affected by natural immune variations between individuals, which is related to genetic variations, environmental exposure and aging (Patin et al., 2018). A variable immune response is crucial in the context of patient-specific models of the immune response, but it might obscure the effects related to the mechanisms under investigation. Therefore, in search of a stable phenotype, human cell lines are an attractive solution for many *in vitro* experiments aiming to validate *in silico* models. As an example, THP-1 is an established monocytic cell line isolated from a patient affected by acute monocytic leukaemia (Tsuchiya et al., 1980). Compared to human primary monocytes, THP-1 cells can be cultured *in vitro* for an almost indefinite time, while maintaining monocytic characteristics. On top of that, there is limited genetic variation between THP-1 cells, thus their phenotype is stable during culture. Nevertheless, the polarization profile of THP-1 cells does not coincide with the one of primary monocytes isolated from PBMCs. It is suggested to use THP-1 cells to validate *in silico* models involving phagocytosis and M1 activation (Shiratori et al., 2017).

#### 4.1.2 Mono-Culture vs. Co-Culture

Single immune cell types have been extensively investigated in traditional mono-culture systems such as tissue culture plastics. Macrophages, for example, are routinely derived from monocytes and activated using standard activators, such as IL-4, IL-10, TGF-β, interferon gamma (IFN-γ) and lipopolysaccharide (LPS). Each standard activator, or their combination, is associated with a specific activation state within the M1-M2 spectrum, identified by specific markers (Murray et al., 2014). Once isolated and seeded on well plates, macrophages can be used as models of the inflammatory response and as phagocytosis assay (Fraser et al., 2009; Westman et al., 2020). As for the *in vitro* mono-culture of SPCs, well plates are routinely used to culture cells and evaluate properties such as cellular proliferation, differentiation, metabolism and senescence following standard protocols (Groeneveldt et al., 2020). Co-culture systems study the interaction between the immune cells and SPCs to model the inflammatory response in bone healing. Traditional co-culture systems consist of both direct co-culture, where cells are in direct contact with each other on cell culture plastics, and indirect co-culture, where transwell inserts are added to culture plates to keep the two cell types separated from each other (Goers et al., 2014). By tuning the pore size of the transwell insert, indirect co-culture models were employed to study the paracrine cell-cell signaling (pore size 0.4 μm, Zhang et al., 2017) or the chemoattractant effect of immune cells on SPCs (pore size 8 μm, Anton et al., 2012). Recent reviews discuss *in vitro* models of the interaction between SPCs and T cells (Kovach et al., 2015) or macrophages (Maruyama et al., 2020), as well as their implications for bone healing.

#### 4.1.3 Advanced *in vitro* Models

Besides traditional cell culture plastics, novel *in vitro* systems enable higher control of the culture environment and cellular interaction. Recent developments of the organ-on-chip technology provide confined engineered microenvironments where biochemical and physical stimuli can be finely tuned over space and time (Zhang et al., 2018). By changing the culture chambers design, organ-on-chips can incorporate multiple cell types cultured both in 2D, as the endothelial monolayer (Del Amo et al., 2016), or in 3D, as cells embedded in a hydrogel (Nasello et al., 2020). The optical transparency of organ-on-chip devices facilitates live cell imaging and monitoring. Compared to traditional transwell inserts, organ-on-chips provide a more physiological environment to study the transendothelial migration of inflammatory cells (Han et al., 2012) and the recruitment of SPCs (Eng et al., 2013). In addition, these systems facilitate the application of both chemical (Moreno-Arotzena et al., 2014) and mechanical cues (Middleton et al., 2017) during culture (Occhetta et al., 2019). Therefore, the combination of inflammation-on-chip (Irimia and Wang, 2018) and bone-on-chip (Nasello et al., 2021) would offer a unique alternative to validate *in silico* models of bone healing by replicating key cellular and environmental interactions of the inflammatory phase.

### 4.2 *In vivo* Models

Despite ethical concerns, validation with animal models is still an essential step for any preclinical study of both the immune system (Wagar et al., 2018) and the bone repair process (Mills and Simpson, 2012; Lammens et al., 2021). Based on the size, *in vivo* models are generally divided into small and large animals. Their use depends on the biological process under investigation and the translational stage of the study (Figure 3A). Here, we discuss the most common small and large animal models used when focusing on the role of the immune system in bone fracture healing. The main results are commented from the modeler’s perspective, in view of creating *in silico* counterparts of these studies. When modelers retrospectively collect data from animal studies to estimate input parameters, they should be aware of the physiological differences between anatomical regions of the skeleton. Besides differences in developmental origins, structural variations in bone composition and direct changes in the biomechanical environment (Cointry et al., 2016), there are regional specializations in cellular composition and differentiation potential. For example, the differentiation potential of skeletal progenitor cells varies between different anatomical sites both in small and large animals (Groeneveldt et al., 2020; Sivaraj et al., 2021).

#### 4.2.1 Small Animal Models

Murine models are widely used to study human diseases and physiology. Despite the differences in the immune system (Mestas and Hughes, 2004) and in the fracture healing process (Haffner-Luntzer et al., 2016) of rodents and humans, murine models can provide clinically relevant results. For instance, murine models were used to validate the clinical observation that fracture healing rate is correlated to higher levels of CD8^+^ T cells in the peripheral blood (Reinke et al., 2013). By depleting or introducing CD8^+^ T cells in a mouse model, the authors observed that fracture regeneration was enhanced or impaired, respectively. Therefore, when modeling the immune effect on bone healing, the levels of CD8^+^ T cells in peripheral blood might be used as a marker of the patient-specific immune reactivity (Reinke et al., 2013).

As for the murine model choice, conventional mouse inbred strains are commonly used since animals share an almost identical genotype, thus leading to higher consistency in the experimental results (Wagar et al., 2018). The lack of genetic variability between individuals is the reason why researchers prefer inbred strains to investigate the fundamental effects of the inflammatory response during fracture healing. The depletion of specific immune cell types, such as macrophages (Schlundt et al., 2018) and T cells (Reinke et al., 2013), was assessed in the mouse inbred strain named C57BL/6N. Moreover, the same inbred strain was used to demonstrate that T and B cells invade the fracture site during the inflammatory phase and the callus mineralization (Könnecke et al., 2014).

#### 4.2.2 Large Animal Models

Large animal models are the most realistic experimental models of human biology and therefore an essential pre-clinical step in translational research (Ribitsch et al., 2020). While nonhuman primates are the most representative model of the human immune system (Wagar et al., 2018), pigs and sheep are normally used to model bone repair since their bone anatomy, mineral composition, regeneration capacity and biomechanical properties are relatively similar to human’s (Sparks et al., 2020). Moreover, compared to mice, their immune system is closer to the human one (Lüthje et al., 2018). As a consequence, pigs and sheep are the first choices as large animal models of the inflammatory response in bone fracture healing.

Compared to small animal models, the biological responses of large animal models are more heterogeneous. While small animal models mostly provide mechanistic insights, such as the effect of depleting a specific cell type, research using large animal models tends to explore the complete biological response and the effects on the entire organism, namely the systemic effects. An *in vivo* study on pigs showed temporal differences in the upregulation of pro-inflammatory cytokines at the fracture site and in the peripheral blood (Horst et al., 2015). Therefore, the validation of *in silico* models using the cytokine levels in blood as input is intrinsically related to a large animal study.

Another key advantage of using large animal models is the possibility to apply clinically relevant mechanical loads to the fracture site. Schmidt-Bleek et al. (2012) showed that mechanical loads delaying bone healing corresponded to a higher presence of T cells in the fracture site, a prolonged inflammatory signaling in the periosteum and reduced angiogenesis. Hence, large animal models should be chosen to assess the interplay between the immune system, bone repair and mechanical loads.

### 4.3 Laboratory Techniques for Experimental Evaluation

Experimental cell-scale techniques can validate *in silico* models describing cellular functions and their response to external stimuli. Therefore, this subsection discusses the quantification of molecular mechanisms behind cell processes which could be applied to both *in vitro* and *in vivo* experiments. Additionally, live imaging techniques are discussed to calibrate cell invasion parameters with *in vitro* migration assays.

As for extracting tissue-level information, standard imaging methods consist of micro-computer tomography (micro-CT), histology and immunohistochemistry. While their use in preclinical models of bone defects has been recently described elsewhere (Sparks et al., 2020), the present subsection shows examples of tissue-level data extracted from images that could validate *in silico* models.

#### 4.3.1 Cell-Scale Techniques

The structural and functional characterization of biological molecules belongs to the scientific fields named *omics*. To explore cellular processes in bone biology, omics technologies characterize, among others, DNA modifications (epigenomics), RNA transcriptions (transcriptomics), protein synthesis (proteomics) and metabolic activity (metabolomics) (Reppe et al., 2017). Transcriptomics, proteomics and metabolomics provide a direct measure of cell survival, proliferation, differentiation and phenotype (Calciolari and Donos, 2020). Therefore, omics technologies can validate *in silico* models of bone healing by coupling cellular function to tissue adaptation (Figure 3B).

Regarding transcriptomics, RNA sequencing (RNA-seq) technologies measure whole transcriptomes, thus they simultaneously analyze the gene expression profile of thousands of genes (Stark et al., 2019; Calciolari and Donos, 2020). When applied to fracture healing, RNA-seq revealed differences in gene expression associated to skeletal and vascular formation between two mice strains, which was correlated to differences in endochondral bone formation (Grimes et al., 2011). Full and stress fractures revealed different transcriptional profiles during repair, with higher expression of inflammatory and immune-related genes in full fractures (Coates et al., 2019). In addition, RNA-seq can measure the dynamic changes in the transcriptome. For example, RNA-seq showed that bone marrow stromal cells upregulate pro-inflammatory gene expression during aging, supporting the hypothesis of a regulatory effect on hematopoietic stem cells (Helbling et al., 2019).

As for proteomics, multiplex immunoassays use specific detection antibodies to measure the level of target proteins. Therefore, immunoassays can quantify a large set of inflammatory cytokines from serum or hematoma samples (Horst et al., 2015), as well as cytokines and osteogenic factors synthesized by macrophages and osteoprogenitor cells *in vitro* (Zhang et al., 2017). Another analytical tool in proteomics is mass spectrometry, which can be used to evaluate the protein composition of the SPC secretome and its variation in a pro-inflammatory environment (Maffioli et al., 2017).

Concerning metabolomics, cell metabolism is continuously altered in bone healing (Loeffler et al., 2018) and experimental techniques can measure both the metabolites produced and the metabolic pathway activities. Glucose and lactate levels are routinely measured in cell culture media, while glucose consumption and lactate secretion can be calculated by comparison with the unspent medium. An increase in glucose uptake and lactate secretion is associated with the M1 macrophages (Galván-Peña and O’Neill, 2014), but their direct calculation from the cell culture media cannot be related to the specific metabolic pathway producing lactate from glucose. Variations in metabolite levels in the media might be related to faster uptake or lower secretion, rather than to a switch in the metabolic routes. To quantify the activity of each metabolic pathway, glucose is labeled with isotope tracers and incorporated radioactivity is measured. Therefore, isotope tracing reveals differences in glucose uptake for M1 and M2 macrophages (Vats et al., 2006).

It is important to mention that traditional omics technologies consist of analyzing the bulk sample, meaning that the quantified data refers to the whole cell population or tissue. Therefore, bulk omics technologies lose the information regarding RNA transcription, protein synthesis and metabolic activity of individual cells. To maintain the biological information of individual cells, novel advances in the omics field separate and analyze single cells from the population (Barh and Azevedo, 2019). For example, high-throughput techniques for RNA-seq allow to measure the whole transcriptome of single cells (scRNA-seq) (Goodwin et al., 2016). By using multiplexed and parallel detection systems, scRNA-seq is generating data that can be used to construct cell atlases from animal and human tissues, both from pathological and physiological conditions (Camp et al., 2018). Although the majority of scRNA-seq methods do not preserve the spatial information of transcriptomic data, novel methods are arising to first localize cells in tissue sections and then sequence RNA (Camp et al., 2018). It is clear that the calibration and validation of *in silico* models, especially discrete models, would benefit from cell atlases reporting the variations of the transcriptome in bone fractures over space and time.

Besides omics technologies to quantify the molecular mechanisms, migration assays are relevant experimental techniques to measure cell-scale parameters in bone healing. For instance, cell culture inserts can assess SPC migration *in vitro* and already quantified higher migration capacity under inflammatory conditions (Anton et al., 2012). However, the *in vitro* environment of a cell culture insert does not represent the 3D extracellular matrix in which cells are embedded *in vivo*. In search of a more representative structural and biological environment, organ-on-chip systems offer confined 3D culture chambers to monitor the migration of inflammatory and osteoprogenitor cells throughout the experiment (Del Amo et al., 2018; Irimia and Wang, 2018). By tuning the microstructural properties in the culture chamber, such as using fibrin or collagen hydrogels, the organ-on-chip can mimic the different extracellular matrices during bone repair. By changing the cell types and the mechano-chemical stimuli in the device, the organ-on-chip can selectively identify the role of different factors in cell migration. Therefore, *in silico* modelers can use cell migration assays in organ-on-chips both to calibrate and validate their predictions.

#### 4.3.2 Tissue-Scale Techniques

Experimental methods to evaluate bone repair at tissue level are widely applied to animal studies and they mostly rely on imaging techniques. Imaging techniques provide qualitative and quantitative information about the analyzed tissue, thus they can validate bone healing *in silico* models (Figure 3B). For example, micro-CT provides metric and non-metric parameters of the bone tissue, such as the mineral density of the bone matrix and trabecular morphology (Müller, 2009). Micro-CT imaging has been applied recently for the non-invasive monitoring of fracture healing in mice. By registering time-lapsed scans of the fracture, micro-CT facilitates the assessment of bone parameters throughout the healing process, without altering the callus properties (Wehrle et al., 2019). This imaging technique has already been coupled to FE models of the mechanical *in vivo* environment, as it substantially contributed to the creation of a personalized bone regeneration model (Tourolle né Betts et al., 2020). Moreover, the combination of micro-CT images and FE models reveals the influence of mechanics on the processes of bone formation and resorption (Birkhold et al., 2014), which can be used to validate *in silico* models of bone adaptation (Schulte et al., 2013).

While micro-CT imaging allows to quantify the newly formed mineralized tissue, histological sections provide histomorphological parameters of the regenerated bone. The histomorphometrical analysis of the Movat Pentachrome staining quantifies the relative area of bone marrow and connective, cartilaginous and osseous tissue (Schlundt et al., 2018). Additionally, immunohistochemical analyses can be used to stain specific cells in a tissue and quantify their density, thus they can identify the different cell types within the fracture site. The output of immunohistochemical analyses is the fraction of the target cell type, such as CD8^+^ T cells, M1 macrophages or osteoblasts, in the stained section (Wendler et al., 2019).

## 5 Towards the Next Generation of Bone Healing *in silico* Models

Considering all the computational models of bone healing that have been developed in the last years, it is surprising that almost none of them describe the early inflammatory phase. As the initiator of the bone healing process, inflammation has a considerable impact in the later stages of bone repair: if the inflammatory response is too strongly down- or upregulated, the fracture can result in non-union. To our knowledge, only one *in silico* model of the inflammatory response in bone healing was developed, which captured the effect of pro- and anti-inflammatory pathways on the healing outcome (Kojouharov et al., 2017; Trejo et al., 2019). Although this model laid a strong foundation within the field of computational bone fracture healing, it still has limitations, among them the lack of spatial distribution of cells and cytokines within the healing region. Therefore, this review aims to guide the design and the validation of the next generation of bone fracture healing *in silico* models, which will include the inflammatory phase.

The biological problem was initially defined by exploring the process of bone healing, with particular attention to the inflammatory phase and its cellular and subcellular components. The inflammatory reaction after bone fracture is a highly complex process, as there is an interplay between different levels (tissue, cellular and subcellular) and systems (musculoskeletal and immune). However, while the mechano-biological activities at cellular and subcellular levels are usually challenging to investigate experimentally, the *in silico* approach can be employed to unveil the hidden events happening at different time and length scales. Following the current trend of developing hybrid multiscale models (Checa et al., 2011; Carlier et al., 2012; Ceresa et al., 2018; Borgiani et al., 2019) to integrate individual (sub)cellular contributions to tissue dynamics, it seems straightforward that the computational research of the inflammatory phase in bone healing should take advantage from similar methodologies. Hybrid multiscale models benefit from both continuous models, which capture the mechano-regulation of tissue and cellular dynamics, and discrete models, which describe the stochastic interactions at cellular and subcellular level occurring during the immune response.

At the current state-of-the-art, numerous *in silico* models of the inflammatory response for different organ systems have been developed within the field of computational immunology (see Subsection 3.2). Since the inflammatory response always tends to follow an analogous cascade of events, these *in silico* models generate the basis to simulate the inflammatory phase in bone healing. Within computational immunology there is a clear preference to use discrete approaches, such as agent-based models or cellular automata, to represent the stochasticity of the immune system. Discrete models can capture better the cascade of cells and subcellular factors that characterize the inflammatory response in bone healing at different time and length scales. However, continuous algorithms capture the dynamics of tissue formation during the subsequent repair and remodeling phases. The development of a comprehensive model that can simulate the mechano-regulation of tissues and the dynamics of large cell populations, while accounting for the probabilistic rules dominating the biological events at subcellular level, requires the combination of continuous and discrete models. For this reason, we believe that the next generation of *in silico* bone healing models will rely on hybrid approaches to include inflammatory regulation.

In order to guarantee the credibility of *in silico* models results in a clearly defined context of use, verification, validation and uncertainty quantification analysis (VVUQ) (ASME, 2018; Parvinian et al., 2019) are essential. Verification ensures the accuracy of the model implementation and validation confirms the correspondence between simulation results and experimental reality. The correspondence between computational outputs and physical reality is intrinsically related to *in vitro* and *in vivo* experiments; as the *in silico* modeling of biological processes, like the ones listed in Table 1, requires thorough parameter estimation. Computational modelers should take advantage of different experimental setups able to provide data for the time and length scales simulated. Table 1 presents a proposal to validate certain biological activities happening during the bone healing process using *in vitro* techniques to replicate specific biological mechanisms in a laboratory, thus providing quantitative data to estimate model parameters. In general, *in vitro* models and multi-omics approaches can validate *in silico* models describing signaling pathways involved in cell fate decision or the response of different cell types under external cues. Therefore, *in vitro* models and omics approaches are highly recommended for the validation of discrete computational models simulating biological events at cellular or subcellular levels. *In vivo* models and imaging techniques are more suitable to validate continuous or hybrid *in silico* models describing the biological response at higher scales, such as histomorphometrical parameters or tissue mineralization. As a result, multiscale or hybrid models covering different time and length scales might require both *in vitro* and *in vivo* models, as well as both cell and tissue level experimental techniques, for their validation. Furthermore, *in vitro* experimental studies are performed to calibrate *in silico* models during their design. The possibility to isolate single biological mechanisms *in vitro* and introduce them as calibrated parameters within the *in silico* model allows to simulate behaviors that resemble the ones observed experimentally and investigate their role in the overall outcome of the simulation. The impact of each parameter on the simulation can be quantified with uncertainty quantification methods.

Uncertainty quantification is tested with sensitivity analyses using e.g. Design of Experiments (DOE) or Machine Learning approaches (Mehrian et al., 2018) to assess whether the uncertainty in model assumptions and parameter values does not lead to non-physiological results. The use of these methodologies to investigate and evaluate the inference of the different parameters can result in valuable information about the most realistic values to describe mechano-biological events. For instance, Isaksson et al. (2008) used DOE to evaluate the significance of multiple factors in bone fracture healing. Parametric uncertainty was addressed by evaluating the outcome of different experiments (simulations, as the study was performed *in silico*) characterized by organized combinations of parametric values assigned to the factors that describe the bone fracture healing process at cellular level (Isaksson et al., 2008). Another class of optimization techniques is the one used by Steiner et al. (2013), namely evolutionary computation. They calibrated their *in silico* model by using the Particle Swarm Optimization (PSO) method to achieve the optimal characterization of the mechanical properties of the tissues in a bone fracture healing scenario. The PSO algorithm evaluated combinations of parameters equally distributed in a stochastic way to find the best combination to describe the tissue mechanics (Steiner et al., 2013). Machine Learning techniques can also be used to evaluate the best value fitting of specific parameters or to categorize certain outputs. An Artificial Neural Network was used by Cilla et al. (2017) to evaluate the geometrical features for the design of a patient-specific short-stem hip implant to contrast the mechanical side effect of prosthetic stress-shielding (Cilla et al., 2017).

The inflammatory response in bone fracture healing has a noteworthy complexity, but *in silico* models help us to understand the principles regulating the diverse events occurring at tissue, cellular and subcellular level. Certainly, the experimental validation of such *in silico* models is mandatory if we aim to go from bench to bedside. With this review, we aimed to highlight the potential of using multiscale *in silico* approaches to tackle bone healing intricacy. Based on the current state-of-the-art, we conclude that hybrid models are particularly suited to simulate adequately the multiscale course of events of the inflammatory phase and its overall role in the healing outcome. We furthermore described possible *in vitro* and *in vivo* methodologies that can be employed to experimentally calibrate the parametric description of the *in silico* model during its development and, afterwards, to validate the computational results and support their bench to bed transition. We believe that the next generation of *in silico* models of bone regeneration should account for inflammatory events to guarantee a more realistic investigation of the process, favoring its employment within a clinical context.

## References

[B1] Adams S. Wuescher L. M. Worth R. Yildirim-Ayan E. (2019). Mechano-Immunomodulation: Mechanoresponsive Changes in Macrophage Activity and Polarization. Ann. Biomed. Eng. 47, 2213–2231. 10.1007/s10439-019-02302-4 31218484 PMC7043232

[B2] Alber M. Chen N. Glimm T. Lushnikov P. M. (2006). Multiscale Dynamics of Biological Cells with Chemotactic Interactions: From a Discrete Stochastic Model to a Continuous Description. Phys. Rev. E 73, 1–11. 10.1103/PhysRevE.73.051901 16802961

[B3] Alexander K. A. Chang M. K. Maylin E. R. Kohler T. Müller R. Wu A. C. (2011). Osteal Macrophages Promote *In Vivo* Intramembranous Bone Healing in a Mouse Tibial Injury Model. J. Bone Mineral Res. 26, 1517–1532. 10.1002/jbmr.354 21305607

[B4] Amini A. R. Laurencin C. T. Nukavarapu S. P. (2012). Bone Tissue Engineering: Recent Advances and Challenges. Crit. Reviews^TM^ Biomed. Eng. 40, 363–408. 10.1615/critrevbiomedeng.v40.i5.10 PMC3766369 23339648

[B5] Anderson A. R. Chaplain M. A. (1998). Continuous and Discrete Mathematical Models of Tumor-Induced Angiogenesis. Bull. Math. Biol. 60, 857–899. 10.1006/bulm.1998.0042 9739618

[B6] Anton K. Banerjee D. Glod J. (2012). Macrophage-Associated Mesenchymal Stem Cells Assume an Activated, Migratory, Pro-inflammatory Phenotype with Increased IL-6 and CXCL10 Secretion. PLoS ONE 7, e35036. 10.1371/journal.pone.0035036 22496888 PMC3319627

[B7] Baht G. S. Vi L. Alman B. A. (2018). The Role of the Immune Cells in Fracture Healing. Curr. Osteoporos. Rep. 16, 138–145. 10.1007/s11914-018-0423-2 29508143 PMC5866272

[B8] Bailón-Plaza A. Van Der Meulen M. C. (2001). A Mathematical Framework to Study the Effects of Growth Factor Influences on Fracture Healing. J. Theor. Biol. 212, 191–209. 10.1006/jtbi.2001.2372 11531385

[B9] Bailón-Plaza A. Van Der Meulen M. C. (2003). Beneficial Effects of Moderate, Early Loading and Adverse Effects of Delayed or Excessive Loading on Bone Healing. J. Biomech. 36, 1069–1077. 10.1016/S0021-9290(03)00117-9 12831731

[B10] Ballotta V. Driessen-Mol A. Bouten C. V. Baaijens F. P. (2014). Strain-dependent Modulation of Macrophage Polarization within Scaffolds. Biomaterials 35, 4919–4928. 10.1016/j.biomaterials.2014.03.002 24661551

[B11] Barh D. Azevedo V. (2019). “Single-Cell Omics,” in Technological Advances and Applications (Cambridge, MA: Academic Press), 1. 10.1016/C2017-0-02420-5

[B12] Barnes G. L. Kostenuik P. J. Gerstenfeld L. C. Einhorn T. A. (1999). Growth Factor Regulation of Fracture Repair. J. Bone Mineral Res. 14, 1805–1815. 10.1359/jbmr.1999.14.11.1805 10571679

[B13] Bastian O. Pillay J. Alblas J. Leenen L. Koenderman L. Blokhuis T. (2011). Systemic Inflammation and Fracture Healing. J. Leukoc. Biol. 89, 669–673. 10.1189/jlb.0810446 21208896

[B14] Bastian O. W. Koenderman L. Alblas J. Leenen L. P. Blokhuis T. J. (2016). Neutrophils Contribute to Fracture Healing by Synthesizing Fibronectin+ Extracellular Matrix Rapidly after Injury. Clin. Immunol. 164, 78–84. 10.1016/j.clim.2016.02.001 26854617

[B15] Bentley K. Gerhardt H. Bates P. A. (2008). Agent-based Simulation of Notch-Mediated Tip Cell Selection in Angiogenic Sprout Initialisation. J. Theor. Biol. 250, 25–36. 10.1016/j.jtbi.2007.09.015 18028963

[B16] Birkhold A. I. Razi H. Duda G. N. Weinkamer R. Checa S. Willie B. M. (2014). The Influence of Age on Adaptive Bone Formation and Bone Resorption. Biomaterials 35, 9290–9301. 10.1016/j.biomaterials.2014.07.051 25128376

[B17] Bittersohl H. Steimer W. (2016). “Intracellular Concentrations of Immunosuppressants,” in Personalized Immunosuppression in Transplantation (Amsterdam, Netherlands: Elsevier), 199–226. 10.1016/B978-0-12-800885-0.00009-6

[B18] Borgiani E. Duda G. Willie B. Checa S. (2015). Bone Healing in Mice: Does it Follow Generic Mechano-Regulation Rules? Facta Universitatis, Ser. Mech. Eng. 13, 217–227.

[B19] Borgiani E. Duda G. N. Checa S. (2017). Multiscale Modeling of Bone Healing: Toward a Systems Biology Approach. Front. Physiol. 8, 287. 10.3389/fphys.2017.00287 28533757 PMC5420595

[B20] Borgiani E. Figge C. Kruck B. Willie B. M. Duda G. N. Checa S. (2019). Age-Related Changes in the Mechanical Regulation of Bone Healing Are Explained by Altered Cellular Mechanoresponse. J. Bone Mineral Res. 34, 1923–1937. 10.1002/jbmr.3801 31121071

[B21] Borgiani E. Duda G. N. Willie B. M. Checa S. (2021). Bone Morphogenetic Protein 2-induced Cellular Chemotaxis Drives Tissue Patterning during Critical-Sized Bone Defect Healing: an In Silico Study. Biomech. Model. Mechanobiol. 20, 1627–1644. 10.1007/s10237-021-01466-0 34047890 PMC8298257

[B22] Borgström F. Karlsson L. Ortsäter G. Norton N. Halbout P. Cooper C. (2020). Fragility Fractures in Europe: Burden, Management and Opportunities. Arch. Osteoporos. 15, 1–21. 10.1007/s11657-020-0706-y PMC7166207 32306163

[B23] Bouchery T. Harris N. (2019). Neutrophil–macrophage Cooperation and its Impact on Tissue Repair. Immunol. Cel Biol. 97, 289–298. 10.1111/imcb.12241 30710448

[B24] Boussommier-Calleja A. Li R. Chen M. B. Wong S. C. Kamm R. D. (2016). Microfluidics: A New Tool for Modeling Cancer–Immune Interactions. Trends Cancer 2, 6–19. 10.1016/j.trecan.2015.12.003 26858990 PMC4743529

[B25] Britton O. J. Bueno-Orovio A. Van Ammel K. Lu H. R. Towart R. Gallacher D. J. (2013). Experimentally Calibrated Population of Models Predicts and Explains Intersubject Variability in Cardiac Cellular Electrophysiology. Proc. Natl. Acad. Sci. U S A. 110, E2098–E2105. 10.1073/pnas.1304382110 23690584 PMC3677477

[B26] Brown B. Price I. Toapanta F. DeAlmeida D. Wiley C. Ross T. (2011). An Agent-Based Model of Inflammation and Fibrosis Following Particulate Exposure in the Lung. Math. Biosci. 231, 186–196. 10.1016/j.mbs.2011.03.005 21385589 PMC3088650

[B27] Burke D. P. Kelly D. J. (2012). Substrate Stiffness and Oxygen as Regulators of Stem Cell Differentiation during Skeletal Tissue Regeneration: A Mechanobiological Model. PLoS One 7, e40737. 10.1371/journal.pone.0040737 22911707 PMC3404068

[B28] Byrne D. P. Lacroix D. Prendergast P. J. (2011). Simulation of Fracture Healing in the Tibia: Mechanoregulation of Cell Activity Using a Lattice Modeling Approach. J. Orthop. Res. 29, 1496–1503. 10.1002/jor.21362 21462249

[B29] Calciolari E. Donos N. (2020). Proteomic and Transcriptomic Approaches for Studying Bone Regeneration in Health and Systemically Compromised Conditions. Proteomics – Clin. Appl. 14, 1900084. 10.1002/prca.201900084 32131137

[B30] Camp J. G. Wollny D. Treutlein B. (2018). Single-cell Genomics to Guide Human Stem Cell and Tissue Engineering. Nat. Methods 15, 661–667. 10.1038/s41592-018-0113-0 30171231

[B31] Carano R. A. Filvaroff E. H. (2003). Angiogenesis and Bone Repair. Drug Discov. Today 8, 980–989. 10.1016/S1359-6446(03)02866-6 14643161

[B32] Carlier A. Geris L. Bentley K. Carmeliet G. Carmeliet P. van Oosterwyck H. (2012). MOSAIC: A Multiscale Model of Osteogenesis and Sprouting Angiogenesis with Lateral Inhibition of Endothelial Cells. PLoS Comput. Biol. 8, e1002724. 10.1371/journal.pcbi.1002724 23071433 PMC3469420

[B33] Carlier A. Geris L. van Gastel N. Carmeliet G. Oosterwyck H. V. (2015). Oxygen as a Critical Determinant of Bone Fracture Healing-A Multiscale Model. J. Theor. Biol. 365, 247–264. 10.1016/j.jtbi.2014.10.012 25452136

[B34] Carlier A. Skvortsov G. A. Hafezi F. Ferraris E. Patterson J. Koc B. (2016). Computational Model-Informed Design and Bioprinting of Cell-Patterned Constructs for Bone Tissue Engineering. Biofabrication 8, 025009. 10.1088/1758-5090/8/2/025009 27187017

[B35] Carter D. R. Beaupré G. S. (1998). Mechanobiology of Skeletal Regeneration. Clin. Orthop. Relat. Res., S41–S55. 10.1097/00003086-199810001-00006 9917625

[B36] Carter D. R. Blenman P. R. Beaupré G. S. (1988). Correlations between Mechanical Stress History and Tissue Differentiation in Initial Fracture Healing. J. Orthop. Res. 6, 736–748. 10.1002/jor.1100060517 3404331

[B37] Celada F. Seiden P. E. (1992). A Computer Model of Cellular Interactions in the Immune System. Immunol. Today 13, 56–62. 10.1016/0167-5699(92)90135-T 1575893

[B38] Ceresa M. Olivares A. L. Noailly J. Ballester M. A. (2018). Coupled Immunological and Biomechanical Model of Emphysema Progression. Front. Physiol. 9, 2712–2715. 10.3389/fphys.2018.00388 PMC5917021 29725304

[B39] Chaplin D. D. (2010). Overview of the Immune Response. J. Allergy Clin. Immunol. 125, S345. 10.1016/j.jaci.2010.01.002 PMC2923430 20176265

[B40] Checa S. Prendergast P. J. Duda G. N. (2011). Inter-species Investigation of the Mechano-Regulation of Bone Healing: Comparison of Secondary Bone Healing in Sheep and Rat. J. Biomech. 44, 1237–1245. 10.1016/j.jbiomech.2011.02.074 21419412

[B41] Cilla M. Borgiani E. Martínez J. Duda G. N. Checa S. (2017). Machine Learning Techniques for the Optimization of Joint Replacements: Application to a Short-Stem Hip Implant. PLoS One 12, 1–16. 10.1371/journal.pone.0183755 PMC5584793 28873093

[B42] Claes L. E. Heigele C. A. (1999). Magnitudes of Local Stress and Strain along Bony Surfaces Predict the Course and Type of Fracture Healing. J. Biomech. 32, 255–266. 10.1016/S0021-9290(98)00153-5 10093025

[B43] Claes L. Recknagel S. Ignatius A. (2012). Fracture Healing under Healthy and Inflammatory Conditions. Nat. Rev. Rheumatol. 8, 133–143. 10.1038/nrrheum.2012.1 22293759

[B44] Coates B. A. McKenzie J. A. Buettmann E. G. Liu X. Gontarz P. M. Zhang B. (2019). Transcriptional Profiling of Intramembranous and Endochondral Ossification after Fracture in Mice. Bone 127, 577–591. 10.1016/j.bone.2019.07.022 31369916 PMC6708791

[B45] Cointry G. R. Nocciolino L. Ireland A. Hall N. M. Kriechbaumer A. Ferretti J. L. (2016). Structural Differences in Cortical Shell Properties between Upper and Lower Human Fibula as Described by pQCT Serial Scans. A Biomechanical Interpretation. Bone 90, 185–194. 10.1016/j.bone.2016.06.007 27302664

[B46] Coquim J. Clemenzi J. Salahi M. Sherif A. Avval P. T. Shah S. (2018). Biomechanical Analysis Using FEA and Experiments of Metal Plate and Bone Strut Repair of a Femur Midshaft Segmental Defect. Biomed. Res. Int. 2018, 4650308. 10.1155/2018/4650308 30420962 PMC6211160

[B47] Dagur P. K. McCoy J. P. (2015). Collection, Storage, and Preparation of Human Blood Cells. Curr. Protoc. Cytometry 73, 5.1.1–5.1.16. 10.1002/0471142956.cy0501s73 PMC4524540 26132177

[B48] Del Amo C. Borau C. Gutiérrez R. Asín J. García-Aznar J. M. (2016). Quantification of Angiogenic Sprouting under Different Growth Factors in a Microfluidic Platform. J. Biomech. 49, 1340–1346. 10.1016/j.jbiomech.2015.10.026 26556715

[B49] Del Amo C. Olivares V. Cóndor M. Blanco A. Santolaria J. Asín J. (2018). Matrix Architecture Plays a Pivotal Role in 3D Osteoblast Migration: The Effect of Interstitial Fluid Flow. J. Mech. Behav. Biomed. Mater. 83, 52–62. 10.1016/j.jmbbm.2018.04.007 29677555

[B50] Dimitriou R. Tsiridis E. Giannoudis P. V. (2005). Current Concepts of Molecular Aspects of Bone Healing. Injury 36, 1392–1404. 10.1016/j.injury.2005.07.019 16102764

[B51] Doblaré M. García J. M. Gómez M. J. (2004). Modelling Bone Tissue Fracture and Healing: A Review. Eng. Fracture Mech. 71, 1809–1840. 10.1016/j.engfracmech.2003.08.003

[B52] Einhorn T. A. (1998). The Cell and Molecular Biology of Fracture Healing. Clin. Orthop. Relat. Res. 355, S7–S21. 10.1097/00003086-199810001-00003 9917622

[B53] Eng G. Lee B. W. Parsa H. Chin C. D. Schneider J. Linkov G. (2013). Assembly of Complex Cell Microenvironments Using Geometrically Docked Hydrogel Shapes. Proc. Natl. Acad. Sci. 110, 4551–4556. 10.1073/pnas.1300569110 23487790 PMC3607001

[B54] Epari D. R. Schell H. Bail H. J. Duda G. N. (2006). Instability Prolongs the Chondral Phase during Bone Healing in Sheep. Bone 38, 864–870. 10.1016/j.bone.2005.10.023 16359937

[B55] Evans S. S. Repasky E. A. Fisher D. T. (2015). Fever and the thermal Regulation of Immunity: The Immune System Feels the Heat. Nat. Rev. Immunol. 15, 335–349. 10.1038/nri3843 25976513 PMC4786079

[B56] Fachada N. Lopes V. Rosa A. (2007). Agent-based Modelling and Simulation of the Immune System: a Review. Epia 2007 Lncs (Lnai) 4874, 300–315.

[B57] Fahy N. Menzel U. Alini M. Stoddart M. J. (2019). Shear and Dynamic Compression Modulates the Inflammatory Phenotype of Human Monocytes *In Vitro* . Front. Immunol. 10, 1–12. 10.3389/fimmu.2019.00383 30891042 PMC6411641

[B58] Faria T. Oliveira J. J. (2020). Global Asymptotic Stability for a Periodic Delay Hematopoiesis Model with Impulses. Appl. Math. Model. 79, 843–864. 10.1016/j.apm.2019.10.063

[B59] Fraser D. A. Laust A. K. Nelson E. L. Tenner A. J. (2009). C1q Differentially Modulates Phagocytosis and Cytokine Responses during Ingestion of Apoptotic Cells by Human Monocytes, Macrophages, and Dendritic Cells. J. Immunol. 183, 6175–6185. 10.4049/jimmunol.0902232 19864605 PMC2843563

[B60] Galván-Peña S. O’Neill L. A. (2014). Metabolic Reprograming in Macrophage Polarization. Front. Immunol. 5, 420. 10.3389/fimmu.2014.00420 25228902 PMC4151090

[B61] García-Aznar J. M. Kuiper J. H. Gómez-Benito M. J. Doblaré M. Richardson J. B. (2007). Computational Simulation of Fracture Healing: Influence of Interfragmentary Movement on the Callus Growth. J. Biomech. 40, 1467–1476. 10.1016/j.jbiomech.2006.06.013 16930609

[B62] Geris L. Gerisch A. Sloten J. V. Weiner R. Oosterwyck H. V. (2008). Angiogenesis in Bone Fracture Healing: A Bioregulatory Model. J. Theor. Biol. 251, 137–158. 10.1016/j.jtbi.2007.11.008 18155732

[B63] Geris L. Sloten J. V. Oosterwyck H. V. (2010). Connecting Biology and Mechanics in Fracture Healing: An Integrated Mathematical Modeling Framework for the Study of Nonunions. Biomech. Model. Mechanobiol. 9, 713–724. 10.1007/s10237-010-0208-8 20333537

[B64] Ghiasi M. S. Chen J. Vaziri A. Rodriguez E. K. Nazarian A. (2017). Bone Fracture Healing in Mechanobiological Modeling: A Review of Principles and Methods. Bone Rep. 6, 87–100. 10.1016/j.bonr.2017.03.002 28377988 PMC5365304

[B65] Gianì F. Russo G. Pennisi M. Sciacca L. Frasca F. Pappalardo F. (2018). Computational Modeling Reveals MAP3K8 as Mediator of Resistance to Vemurafenib in Thyroid Cancer Stem Cells. Bioinformatics 35, 2267–2275. 10.1093/bioinformatics/bty969 30481266

[B66] Gillespie M. T. (2007). Impact of Cytokines and T Lymphocytes upon Osteoclast Differentiation and Function. Arthritis Res. Ther. 9, 7–9. 10.1186/ar2141 PMC1906805 17381830

[B67] Giorgi M. Verbruggen S. W. Lacroix D. (2016). Silico Bone Mechanobiology: Modeling a Multifaceted Biological System. Wiley Interdiscip. Rev. Syst. Biol. Med. 8, 485–505. 10.1002/wsbm.1356 27600060 PMC5082538

[B68] Gómez-Benito M. J. García-Aznar J. M. Kuiper J. H. Doblaré M. (2005). Influence of Fracture gap Size on the Pattern of Long Bone Healing: A Computational Study. J. Theor. Biol. 235, 105–119. 10.1016/j.jtbi.2004.12.023 15833317

[B69] Gómez-Benito M. J. García-Aznar J. M. Kuiper J. H. Doblaré M. (2006). A 3D Computational Simulation of Fracture Callus Formation: Influence of the Stiffness of the External Fixator. J. Biomech. Eng. 128, 290–299. 10.1115/1.2187045 16706578

[B70] Godwin J. W. Pinto A. R. Rosenthal N. A. (2017). Chasing the Recipe for a Pro-regenerative Immune System. Semin. Cel Dev. Biol. 61, 71–79. 10.1016/j.semcdb.2016.08.008 PMC5338634 27521522

[B71] Goers L. Freemont P. Polizzi K. M. (2014). Co-culture Systems and Technologies: Taking Synthetic Biology to the Next Level. J. R. Soc. Interf. 11, 20140065. 10.1098/rsif.2014.0065 PMC4032528 24829281

[B72] Gong C. Milberg O. Wang B. Vicini P. Narwal R. Roskos L. (2017). A Computational Multiscale Agent-Based Model for Simulating Spatio-Temporal Tumour Immune Response to PD1 and PDL1 Inhibition. J. R. Soc. Interf. 14, 20170320. 10.1098/rsif.2017.0320 PMC5636269 28931635

[B73] Goodwin S. McPherson J. D. McCombie W. R. (2016). Coming of Age: Ten Years of Next-Generation Sequencing Technologies. Nat. Rev. Genet. 17, 333–351. 10.1038/nrg.2016.49 27184599 PMC10373632

[B74] Grimes R. Jepsen K. J. Fitch J. L. Einhorn T. A. Gerstenfeld L. C. (2011). The Transcriptome of Fracture Healing Defines Mechanisms of Coordination of Skeletal and Vascular Development during Endochondral Bone Formation. J. Bone Mineral Res. 26, 2597–2609. 10.1002/jbmr.486 21826735

[B75] Groeneveldt L. C. Herpelinck T. Maréchal M. Politis C. van IJcken W. F. J. Huylebroeck D. (2020). The Bone-Forming Properties of Periosteum-Derived Cells Differ between Harvest Sites. Front. Cel Dev. Biol. 8, 554984. 10.3389/fcell.2020.554984 PMC7723972 33324630

[B76] Gruber E. J. Leifer C. A. (2020). Molecular Regulation of TLR Signaling in Health and Disease: Mechano-Regulation of Macrophages and TLR Signaling. Innate Immun. 26, 15–25. 10.1177/1753425919838322 31955624 PMC6974875

[B77] Grundnes O. Reikerås O. (1993). The Importance of the Hematoma for Fracture Healing in Rats. Acta Orthop. Scand. 64, 340–342. 10.3109/17453679308993640 8322595

[B78] Gu Q. Yang H. Shi Q. (2017). Macrophages and Bone Inflammation. J. Orthop. Transl. 10, 86–93. 10.1016/j.jot.2017.05.002 PMC5822954 29662760

[B79] Guyot Y. Papantoniou I. Chai Y. C. Van Bael S. Schrooten J. Geris L. (2014). A Computational Model for Cell/ECM Growth on 3D Surfaces Using the Level Set Method: a Bone Tissue Engineering Case Study. Biomech. Model. Mechanobiol. 13, 1361–1371. 10.1007/s10237-014-0577-5 24696122

[B80] Haffner-Luntzer M. Kovtun A. Rapp A. E. Ignatius A. (2016). Mouse Models in Bone Fracture Healing Research. Curr. Mol. Biol. Rep. 2, 101–111. 10.1007/s40610-016-0037-3

[B81] Han S. Yan J.-J. Shin Y. Jeon J. J. Won J. Eun Jeong H. (2012). A Versatile Assay for Monitoring In Vivo-like Transendothelial Migration of Neutrophils. Lab. A Chip 12, 3861. 10.1039/c2lc40445a 22903230

[B82] Harasymowicz N. S. Rashidi N. Savadipour A. Wu C. L. Tang R. Bramley J. (2021). Single-cell RNA Sequencing Reveals the Induction of Novel Myeloid and Myeloid-Associated Cell Populations in Visceral Fat with Long-Term Obesity. FASEB J. 35, 1–17. 10.1096/fj.202001970R PMC8743141 33566380

[B83] Harwood P. J. Newman J. B. Michael A. L. (2010). (ii) an Update on Fracture Healing and Non-union. Orthop. Trauma 24, 9–23. 10.1016/j.mporth.2009.12.004

[B84] Helbling P. M. Piñeiro-Yáñez E. Gerosa R. Boettcher S. Al-Shahrour F. Manz M. G. (2019). Global Transcriptomic Profiling of the Bone Marrow Stromal Microenvironment during Postnatal Development, Aging, and Inflammation. Cel Rep. 29, 3313–3330.e4. 10.1016/j.celrep.2019.11.004 31801092

[B85] Hoff P. Gaber T. Strehl C. Schmidt-Bleek K. Lang A. Huscher D. (2016). Immunological Characterization of the Early Human Fracture Hematoma. Immunol. Res. 64, 1195–1206. 10.1007/s12026-016-8868-9 27629117

[B86] Hoff P. Gaber T. Strehl C. Jakstadt M. Hoff H. Schmidt-Bleek K. (2017). A Pronounced Inflammatory Activity Characterizes the Early Fracture Healing Phase in Immunologically Restricted Patients. Int. J. Mol. Sci. 18, 583. 10.3390/ijms18030583 PMC5372599 28282868

[B87] Horst K. Eschbach D. Pfeifer R. Hübenthal S. Sassen M. Steinfeldt T. (2015). Local Inflammation in Fracture Hematoma: Results from a Combined Trauma Model in Pigs. Mediators Inflamm. 2015, 126060. 10.1155/2015/126060 25694748 PMC4324980

[B88] Hundsdorfer W. Verwer J. (2003). Numerical Solution of Time-dependent Advection–Diffusion–Reaction Equations, 33 Heidelberg, Germany: Springer-Verlag Berlin Heidelberg. 10.1007/978-3-662-09017-6

[B89] Irimia D. Wang X. (2018). Inflammation-on-a-Chip: Probing the Immune System *Ex Vivo* . Trends Biotechnol. 36, 923–937. 10.1016/j.tibtech.2018.03.011 29728272 PMC6098972

[B90] Isaksson H. Wilson W. van Donkelaar C. C. Huiskes R. Ito K. (2006). Comparison of Biophysical Stimuli for Mechano-Regulation of Tissue Differentiation during Fracture Healing. J. Biomech. 39, 1507–1516. 10.1016/j.jbiomech.2005.01.037 15972212

[B91] Isaksson H. van Donkelaar C. C. Huiskes R. Yao J. Ito K. (2008). Determining the Most Important Cellular Characteristics for Fracture Healing Using Design of Experiments Methods. J. Theor. Biol. 255, 26–39. 10.1016/j.jtbi.2008.07.037 18723028

[B92] Jahn C. Weidinger G. (2017). Regulatory T Cells Know what Is Needed to Regenerate. Dev. Cel. 43, 651–652. 10.1016/j.devcel.2017.12.010 29257945

[B93] Jain N. Moeller J. Vogel V. (2019). Mechanobiology of Macrophages: How Physical Factors Coregulate Macrophage Plasticity and Phagocytosis. Annu. Rev. Biomed. Eng. 21, 267–297. 10.1146/annurev-bioeng-062117-121224 31167103

[B94] Jerez S. Díaz-Infante S. Chen B. (2018). Fluctuating Periodic Solutions and Moment Boundedness of a Stochastic Model for the Bone Remodeling Process. Math. Biosci. 299, 153–164. 10.1016/j.mbs.2018.03.006 29526549

[B95] Karnes J. M. Daffner S. D. Watkins C. M. (2015). Multiple Roles of Tumor Necrosis Factor-Alpha in Fracture Healing. Bone 78, 87–93. 10.1016/j.bone.2015.05.001 25959413

[B96] Klein P. Schell H. Streitparth F. Heller M. Kassi J. P. Kandziora F. (2003). The Initial Phase of Fracture Healing Is Specifically Sensitive to Mechanical Conditions. J. Orthop. Res. 21, 662–669. 10.1016/s0736-0266(02)00259-0 12798066

[B97] Könnecke I. Serra A. El Khassawna T. Schlundt C. Schell H. Hauser A. (2014). T and B Cells Participate in Bone Repair by Infiltrating the Fracture Callus in a Two-Wave Fashion. Bone 64, 155–165. 10.1016/j.bone.2014.03.052 24721700

[B98] Kojouharov H. V. Trejo I. Chen-Charpentier B. M. (2017). “Modeling the Effects of Inflammation in Bone Fracture Healing,” in AIP Conference Proceedings, Albena, Bulgaria, June 21–26, 2017, 1895. 10.1063/1.5007359

[B99] Kolar P. Schmidt-Bleek K. Schell H. Gaber T. Toben D. Schmidmaier G. (2010). The Early Fracture Hematoma and its Potential Role in Fracture Healing. Tissue Eng. - B: Rev. 16, 427–434. 10.1089/ten.teb.2009.0687 20196645

[B100] Kovach T. K. Dighe A. S. Lobo P. I. Cui Q. (2015). Interactions between MSCs and Immune Cells: Implications for Bone Healing. J. Immunol. Res. 2015, 1–17. 10.1155/2015/752510 PMC4427002 26000315

[B101] Kovtun A. Bergdolt S. Wiegner R. Radermacher P. Huber-Lang M. Ignatius A. (2016). The Crucial Role of Neutrophil Granulocytes in Bone Fracture Healing. Eur. Cell Mater. 32, 152–162. 10.22203/eCM.v032a10 27452963

[B102] Kumar R. Clermont G. Vodovotz Y. Chow C. C. (2004). The Dynamics of Acute Inflammation. J. Theor. Biol. 230, 145–155. 10.1016/j.jtbi.2004.04.044 15321710

[B103] Lacroix D. Prendergast P. (2002). A Mechano-Regulation Model for Tissue Differentiation during Fracture Healing: Analysis of gap Size and Loading. J. Biomech. 35, 1163–1171. 10.1016/S0021-9290(02)00086-6 12163306

[B104] Lammens J. Laumen A. Delport H. Vanlauwe J. (2012). The Pentaconcept in Skeletal Tissue Engineering. A Combined Approach for the Repair of Bone Defects. Acta Orthop. Belg. 78, 569–573. 23162950

[B105] Lammens J. Marechal M. Delport H. Geris L. Luyten F. (2021). A Flowchart for the Translational Research of Cell-Based Therapy in the Treatment of Long Bone Defects. J. Regener. Med. 10, 1. 10.37532/jrgm.2021.10(1).175

[B106] Lawson B. A. Drovandi C. C. Cusimano N. Burrage P. Rodriguez B. Burrage K. (2018). Unlocking Data Sets by Calibrating Populations of Models to Data Density: A Study in Atrial Electrophysiology. Sci. Adv. 4. 10.1126/sciadv.1701676 PMC5770172 29349296

[B107] Lisowska B. Kosson D. Domaracka K. (2018). Positives and Negatives of Nonsteroidal Anti-inflammatory Drugs in Bone Healing: The Effects of These Drugs on Bone Repair. Drug Des. Dev. Ther. 12, 1809–1814. 10.2147/dddt.s164565 PMC6016595 29950815

[B108] Liszka T. Orkisz J. (1980). The Finite Difference Method at Arbitrary Irregular Grids and its Application in Applied Mechanics. Comput. Struct. 11, 83–95. Special Issue-Computational Methods in Nonlinear Mechanics. 10.1016/0045-7949(80)90149-2

[B109] Lo C. H. Baratchart E. Basanta D. Lynch C. C. (2020). Computational Modeling Reveals a Key Role for Polarized Myeloid Cells in Controlling Osteoclast Activity during Bone Injury Repair. bioRxiv. 10.1101/2020.10.13.338335 PMC7961065 33723343

[B110] Loeffler J. Duda G. N. Sass F. A. Dienelt A. (2018). The Metabolic Microenvironment Steers Bone Tissue Regeneration. Trends Endocrinol. Metab. 29, 99–110. 10.1016/j.tem.2017.11.008 29290501

[B111] Loi F. Córdova L. A. Pajarinen J. hua Lin T. Yao Z. Goodman S. B. (2016). Inflammation, Fracture and Bone Repair. Bone 86, 119–130. 10.1016/j.bone.2016.02.020 26946132 PMC4833637

[B112] Lüthje F. L. Skovgaard K. Jensen H. E. Kruse Jensen L. (2018). Pigs Are Useful for the Molecular Study of Bone Inflammation and Regeneration in Humans. Lab. Anim. 52, 630–640. 10.1177/0023677218766391 29653496

[B113] Lux T. (2018). Estimation of Agent-Based Models Using Sequential Monte Carlo Methods. J. Econ. Dyn. Control. 91, 391–408. 10.1016/j.jedc.2018.01.021

[B114] Mackey M. Glass L. (1977). Oscillation and Chaos in Physiological Control Systems. Science 197, 287–289. 10.1126/science.267326 267326

[B115] Maffioli E. Nonnis S. Angioni R. Santagata F. Calì B. Zanotti L. (2017). Proteomic Analysis of the Secretome of Human Bone Marrow-Derived Mesenchymal Stem Cells Primed by Pro-inflammatory Cytokines. J. Proteomics 166, 115–126. 10.1016/j.jprot.2017.07.012 28739509

[B116] Malizos K. N. Papatheodorou L. K. (2005). The Healing Potential of the Periosteum: Molecular Aspects. Injury 36, S13–S19. 10.1016/j.injury.2005.07.030 16188544

[B117] Manabe N. Kawaguchi H. Chikuda H. Miyaura C. Inada M. Nagai R. (2001). Connection between B Lymphocyte and Osteoclast Differentiation Pathways. J. Immunol. 167, 2625–2631. 10.4049/jimmunol.167.5.2625 11509604

[B118] Marder E. Taylor A. L. (2011). Multiple Models to Capture the Variability in Biological Neurons and Networks. Nat. Neurosci. 14, 133–138. 10.1038/nn.2735 21270780 PMC3686573

[B119] Marsell R. Einhorn T. A. (2011). The Biology of Fracture Healing. Injury 42, 551–555. 10.1016/j.injury.2011.03.031 21489527 PMC3105171

[B120] Marsh D. (1998). Concepts of Fracture union, Delayed union, and Nonunion. Clin. Orthop. Relat. Res. 355, S22–S30. 10.1097/00003086-199810001-00004 9917623

[B121] Martínez I. V. Gómez E. J. Hernando M. E. Villares R. Mellado M. (2012). Agent-based Model of Macrophage Action on Endocrine Pancreas. Int. J. Data Mining Bioinformatics 6, 355–368. 10.1504/ijdmb.2012.049293 23155767

[B122] Maruyama M. Rhee C. Utsunomiya T. Zhang N. Ueno M. Yao Z. (2020). Modulation of the Inflammatory Response and Bone Healing. Front. Endocrinol. 11, 386. 10.3389/fendo.2020.00386 PMC7325942 32655495

[B123] Maslin C. Kedzierska K. Webster N. Muller W. Crowe S. (2005). Transendothelial Migration of Monocytes: The Underlying Molecular Mechanisms and Consequences of HIV-1 Infection. Curr. HIV Res. 3, 303–317. 10.2174/157016205774370401 16250878

[B124] McWhorter F. Y. Wang T. Nguyen P. Chung T. Liu W. F. (2013). Modulation of Macrophage Phenotype by Cell Shape. Proc. Natl. Acad. Sci. U S A. 110, 17253–17258. 10.1073/pnas.1308887110 24101477 PMC3808615

[B125] Medzhitov R. Janeway C. A. (1997). Innate Immunity : Impact on the Adaptive Immune Response. Health San Francisco 9, 4–9. 10.1016/S0952-7915(97)80152-5 9039775

[B126] Medzhitov R. Janeway C. (2000). Innate Immune Recognition: Mechanisms and Pathways. Immunol Rev. 173, 89–97. 10.1034/j.1600-065x.2000.917309.x 10719670

[B127] Mehrian M. Guyot Y. Papantoniou I. Olofsson S. Sonnaert M. Misener R. (2018). Maximizing Neotissue Growth Kinetics in a Perfusion Bioreactor: an In Silico Strategy Using Model Reduction and Bayesian Optimization. Biotechnol. Bioeng. 115, 617–629. 10.1002/bit.26500 29205280

[B128] Mescher A. L. (2017). Macrophages and Fibroblasts during Inflammation and Tissue Repair in Models of Organ Regeneration. Regeneration 4, 39–53. 10.1002/reg2.77 28616244 PMC5469729

[B129] Mestas J. Hughes C. C. W. (2004). Of Mice and Not Men: Differences between Mouse and Human Immunology. J. Immunol. 172, 2731–2738. 10.4049/jimmunol.172.5.2731 14978070

[B130] Mi Q. Rivière B. Clermont G. Steed D. L. Vodovotz Y. (2007). Agent-based Model of Inflammation and Wound Healing: Insights into Diabetic Foot Ulcer Pathology and the Role of Transforming Growth Factor-*Β*1. Wound Repair Regen. 15, 671–682. 10.1111/j.1524-475X.2007.00271.x 17971013

[B131] Middleton K. Al-Dujaili S. Mei X. Günther A. You L. (2017). Microfluidic Co-culture Platform for Investigating Osteocyte-Osteoclast Signalling during Fluid Shear Stress Mechanostimulation. J. Biomech. 59, 35–42. 10.1016/j.jbiomech.2017.05.012 28552413

[B132] Mills L. A. Simpson A. H. R. W. (2012). *In Vivo* models of Bone Repair. The J. Bone Jt. Surg., Br. vol. 94-B, 865–874. 10.1302/0301-620x.94b7.27370 22733938

[B133] Mills L. A. Aitken S. A. Simpson A. H. R. (2017). The Risk of Non-union Per Fracture: Current Myths and Revised Figures from a Population of over 4 Million Adults. Acta Orthop. 88, 434–439. 10.1080/17453674.2017.1321351 28508682 PMC5499337

[B134] Moore S. R. Saidel G. M. Knothe U. Knothe Tate M. L. (2014). Mechanistic, Mathematical Model to Predict the Dynamics of Tissue Genesis in Bone Defects via Mechanical Feedback and Mediation of Biochemical Factors. PLoS Comput. Biol. 10, e1003604. 10.1371/journal.pcbi.1003604 24967742 PMC4072518

[B135] Moreno-Arotzena O. Mendoza G. Cóndor M. Rüberg T. García-Aznar J. M. (2014). Inducing Chemotactic and Haptotactic Cues in Microfluidic Devices for Three-Dimensional *In Vitro* Assays. Biomicrofluidics 8, 064122. 10.1063/1.4903948 25587374 PMC4265035

[B136] Mosser D. M. Edwards J. P. (2008). Exploring the Full Spectrum of Macrophage Activation. Nat. Rev. Immunol. 8, 958–969. 10.1038/nri2448 19029990 PMC2724991

[B137] Müller R. (2009). Hierarchical Microimaging of Bone Structure and Function. Nat. Rev. Rheumatol. 5, 373–381. 10.1038/nrrheum.2009.107 19568252

[B138] Murray P. J. Allen J. E. Biswas S. K. Fisher E. A. Gilroy D. W. Goerdt S. (2014). Macrophage Activation and Polarization: Nomenclature and Experimental Guidelines. Immunity 41, 14–20. 10.1016/j.immuni.2014.06.008 25035950 PMC4123412

[B139] Murray J. D. (1989). Mathematical Biology, 19 Heidelberg, Germany: Springer-Verlag Berlin Heidelberg. 10.1007/978-3-662-08539-4

[B140] Nagaraja S. Wallqvist A. Reifman J. Mitrophanov A. Y. (2014). Computational Approach to Characterize Causative Factors and Molecular Indicators of Chronic Wound Inflammation. J. Immunol. 192, 1824–1834. 10.4049/jimmunol.1302481 24453259

[B141] Nagatani Y. Imaizumi H. Fukuda T. Matsukawa M. Watanabe Y. Otani T. (2006). Applicability of Finite-Difference Time-Domain Method to Simulation of Wave Propagation in Cancellous Bone. Jpn. J. Appl. Phys. 45, 7186–7190. 10.1143/jjap.45.7186

[B142] Nasello G. Alamán-Díez P. Schiavi J. Pérez M. Á. McNamara L. García-Aznar J. M. (2020). Primary Human Osteoblasts Cultured in a 3D Microenvironment Create a Unique Representative Model of Their Differentiation into Osteocytes. Front. Bioeng. Biotechnol. 8, 336. 10.3389/fbioe.2020.00336 32391343 PMC7193048

[B143] Nasello G. Cóndor M. Vaughan T. Schiavi J. (2021). Designing Hydrogel-Based Bone-On-Chips for Personalized Medicine. Appl. Sci. 11, 4495. 10.3390/app11104495

[B144] Occhetta P. Mainardi A. Votta E. Vallmajo-Martin Q. Ehrbar M. Martin I. (2019). Hyperphysiological Compression of Articular Cartilage Induces an Osteoarthritic Phenotype in a Cartilage-On-A-Chip Model. Nat. Biomed. Eng. 3, 545–557. 10.1038/s41551-019-0406-3 31160722

[B145] Olsen L. Sherratt J. A. Maini P. K. Arnold F. (1997). A Mathematical Model for the Capillary Endothelial Cell-Extracellular Matrix Interactions in Wound-Healing Angiogenesis. IMA J. Math. Appl. Med. Biol. 14, 261–281. 10.1093/imammb/14.4.261 9415995

[B146] OReilly A. Hankenson K. D. Kelly D. J. (2016). A Computational Model to Explore the Role of Angiogenic Impairment on Endochondral Ossification during Fracture Healing. Biomech. Model. Mechanobiol. 15, 1279–1294. 10.1007/s10237-016-0759-4 26825534

[B147] Oryan A. Monazzah S. Bigham-Sadegh A. (2015). Bone Injury and Fracture Healing Biology. Biomed. Environ. Sci. 28, 57–71. 10.3967/bes2015.006 25566863

[B148] Osta B. Benedetti G. Miossec P. (2014). Classical and Paradoxical Effects of TNF-*α* on Bone Homeostasis. Front. Immunol. 5, 1–9. 10.3389/fimmu.2014.00048 24592264 PMC3923157

[B149] Osuka A. Ogura H. Ueyama M. Shimazu T. Lederer J. A. (2014). Immune Response to Traumatic Injury: harmony and Discordance of Immune System Homeostasis. Acute Med. Surg. 1, 63–69. 10.1002/ams2.17 29930824 PMC5997205

[B150] Pajarinen J. Lin T. Gibon E. Kohno Y. Maruyama M. Nathan K. (2019). Mesenchymal Stem Cell-Macrophage Crosstalk and Bone Healing. Biomaterials 196, 80–89. 10.1016/j.biomaterials.2017.12.025 29329642 PMC6028312

[B151] Papantoniou I. Nilsson Hall G. Loverdou N. Lesage R. Herpelinck T. Mendes L. (2021). Turning Nature’s Own Processes into Design Strategies for Living Bone Implant Biomanufacturing: a Decade of Developmental Engineering. Adv. Drug Deliv. Rev. 169, 22–39. 10.1016/j.addr.2020.11.012 33290762 PMC7839840

[B152] Pape H. C. Evans A. Kobbe P. (2010). Autologous Bone Graft: Properties and Techniques. J. orthop. Trauma 24, S36–S40. 10.1097/bot.0b013e3181cec4a1 20182233

[B153] Pappalardo F. Pennisi M. Motta S. (2010). “Universal Immune System Simulator Framework (UISS),” in Proceedings of the First ACM International Conference on Bioinformatics and Computational Biology, Niagara Falls, NY, August 2–4, 2010 (Association for Computing Machinery, BCB ’10), 649–650. 10.1145/1854776.1854900

[B154] Pappalardo F. Russo G. Pennisi M. Parasiliti Palumbo G. A. Sgroi G. Motta S. (2020). The Potential of Computational Modeling to Predict Disease Course and Treatment Response in Patients with Relapsing Multiple Sclerosis. Cells 9, 586. 10.3390/cells9030586 PMC7140535 32121606

[B155] Parvinian B. Pathmanathan P. Daluwatte C. Yaghouby F. Gray R. A. Weininger S. (2019). Credibility Evidence for Computational Patient Models Used in the Development of Physiological Closed-Loop Controlled Devices for Critical Care Medicine. Front. Physiol. 10, 220. 10.3389/fphys.2019.00220 30971934 PMC6445134

[B156] Patin E. Hasan M. Bergstedt J. Rouilly V. Libri V. Urrutia A. (2018). Natural Variation in the Parameters of Innate Immune Cells Is Preferentially Driven by Genetic Factors. Nat. Immunol. 19, 302–314. 10.1038/s41590-018-0049-7 29476184

[B157] Peiffer V. Gerisch A. Vandepitte D. Van Oosterwyck H. Geris L. (2011). A Hybrid Bioregulatory Model of Angiogenesis during Bone Fracture Healing. Biomech. Model. Mechanobiol. 10, 383–395. 10.1007/s10237-010-0241-7 20827500

[B158] Pennisi M. Rajput A. M. Toldo L. Pappalardo F. (2013). Agent Based Modeling of Treg-Teff Cross Regulation in Relapsing-Remitting Multiple Sclerosis. BMC Bioinf. 14 Suppl 16, S9. 10.1186/1471-2105-14-S16-S9 PMC3853330 24564794

[B159] Perier-Metz C. Duda G. N. Checa S. (2020). Mechano-Biological Computer Model of Scaffold-Supported Bone Regeneration: Effect of Bone Graft and Scaffold Structure on Large Bone Defect Tissue Patterning. Front. Bioeng. Biotechnol. 8, 1–15. 10.3389/fbioe.2020.585799 33262976 PMC7686036

[B160] Perren S. M. (2002). Evolution of the Internal Fixation of Long Bone Fractures: the Scientific Basis of Biological Internal Fixation: Choosing a New Balance between Stability and Biology. The J. bone Jt. surg., Br. vol. 84, 1093–1110. 10.1302/0301-620x.84b8.0841093 12463652

[B161] Plouffe B. D. Murthy S. K. Lewis L. H. (2015). Fundamentals and Application of Magnetic Particles in Cell Isolation and Enrichment: a Review. Rep. Prog. Phys. 78, 016601. 10.1088/0034-4885/78/1/016601 25471081 PMC4310825

[B162] Prendergast P. Huiskes R. Soballe K. (1997). Biophysical Stimuli on Cells during Tissue Differentiation at Implant Interfaces. J. Biomech. 30, 539–548. 10.1016/S0021-9290(96)00140-6 9165386

[B163] Prokharau P. A. Vermolen F. J. García-Aznar J. M. (2012). A Mathematical Model for Cell Differentiation, as an Evolutionary and Regulated Process. Comput. Methods Biomech. Biomed. Eng. 17, 1051–1070. 10.1080/10255842.2012.736503 23113617

[B164] Reinke S. Geissler S. Taylor W. R. Schmidt-Bleek K. Juelke K. Schwachmeyer V. (2013). Terminally Differentiated CD8+ T Cells Negatively Affect Bone Regeneration in Humans. Sci. Transl. Med. 5, 177ra36. 10.1126/scitranslmed.3004754 23515078

[B165] Reppe S. Datta H. K. Gautvik K. M. (2017). Omics Analysis of Human Bone to Identify Genes and Molecular Networks Regulating Skeletal Remodeling in Health and Disease. Bone 101, 88–95. 10.1016/j.bone.2017.04.012 28450214

[B166] Reynolds A. Rubin J. Clermont G. Day J. Vodovotz Y. Bard Ermentrout G. (2006). A Reduced Mathematical Model of the Acute Inflammatory Response: I. Derivation of Model and Analysis of Anti-inflammation. J. Theor. Biol. 242, 220–236. 10.1016/j.jtbi.2006.02.016 16584750

[B167] Ribeiro F. O. Gómez-Benito M. J. Folgado J. Fernandes P. R. García-Aznar J. M. (2015). In Silico mechano-chemical Model of Bone Healing for the Regeneration of Critical Defects: The Effect of BMP-2. PLoS One 10, 1–25. 10.1371/journal.pone.0127722 PMC4456173 26043112

[B168] Ribitsch I. Baptista P. M. Lange-Consiglio A. Melotti L. Patruno M. Jenner F. (2020). Large Animal Models in Regenerative Medicine and Tissue Engineering: To Do or Not to Do. Front. Bioeng. Biotechnol. 8, 972. 10.3389/fbioe.2020.00972 32903631 PMC7438731

[B169] Rios F. J. Touyz R. M. Montezano A. C. (2017). “Isolation and Differentiation of Human Macrophages,” in Hypertension (New York, NY: Humana Press), 311–320. 10.1007/978-1-4939-6625-7_24 28116726

[B170] Russo G. Pennisi M. Fichera E. Motta S. Raciti G. Viceconti M. (2020a). In Silico trial to Test COVID-19 Candidate Vaccines: a Case Study with UISS Platform. BMC Bioinf. 21, 527. 10.1186/s12859-020-03872-0 PMC7733700 33308153

[B171] Russo G. Sgroi G. Parasiliti Palumbo G. A. Pennisi M. Juarez M. A. Cardona P.-J. (2020b). Moving Forward through the In Silico Modeling of Tuberculosis: a Further Step with UISS-TB. BMC Bioinf. 21, 458. 10.1186/s12859-020-03762-5 PMC7733696 33308139

[B172] Sadtler K. Estrellas K. Allen B. W. Wolf M. T. Fan H. Tam A. J. (2016). Developing a Pro-regenerative Biomaterial Scaffold Microenvironment Requires T Helper 2 Cells. Science 352, 366–370. 10.1126/science.aad9272 27081073 PMC4866509

[B173] Schlundt C. Schell H. Goodman S. B. Vunjak-Novakovic G. Duda G. N. Schmidt-Bleek K. (2015). Immune Modulation as a Therapeutic Strategy in Bone Regeneration. J. Exp. Orthop. 2, 1–10. 10.1186/s40634-014-0017-6 26914869 PMC4545842

[B174] Schlundt C. El Khassawna T. Serra A. Dienelt A. Wendler S. Schell H. (2018). Macrophages in Bone Fracture Healing: Their Essential Role in Endochondral Ossification. Bone 106, 78–89. 10.1016/j.bone.2015.10.019 26529389

[B175] Schmidt-Bleek K. Schell H. Schulz N. Hoff P. Perka C. Buttgereit F. (2012). Inflammatory Phase of Bone Healing Initiates the Regenerative Healing cascade. Cel Tissue Res. 347, 567–573. 10.1007/s00441-011-1205-7 21789579

[B176] Schmidt-Bleek K. Marcucio R. Duda G. (2016). Future Treatment Strategies for Delayed Bone Healing: An Osteoimmunologic Approach. J. Am. Acad. Orthop. Surg. 24, e134–e135. 10.5435/JAAOS-D-16-00513 27579817 PMC5146983

[B177] Schulte F. A. Zwahlen A. Lambers F. M. Kuhn G. Ruffoni D. Betts D. (2013). Strain-adaptive In Silico Modeling of Bone Adaptation — A Computer Simulation Validated by *In Vivo* Micro-computed Tomography Data. Bone 52, 485–492. 10.1016/j.bone.2012.09.008 22985889

[B178] Seiden P. E. Celada F. (1992). A Model for Simulating Cognate Recognition and Response in the Immune System. J. Theor. Biol. 158, 329–357. 10.1016/s0022-5193(05)80737-4 1287364

[B179] Shi Z. Chapes S. K. Ben-Arieh D. Wu C. H. (2016). An Agent-Based Model of a Hepatic Inflammatory Response to salmonella: A Computational Study under a Large Set of Experimental Data. PLoS One 11, e0161131. 10.1371/journal.pone.0161131 27556404 PMC4996536

[B180] Shiratori H. Feinweber C. Luckhardt S. Linke B. Resch E. Geisslinger G. (2017). THP-1 and Human Peripheral Blood Mononuclear Cell-Derived Macrophages Differ in Their Capacity to Polarize *In Vitro* . Mol. Immunol. 88, 58–68. 10.1016/j.molimm.2017.05.027 28600970

[B181] Sierra M. Miana-Mena F. J. Calvo B. Muñoz M. J. Rodríguez J. F. Grasa J. (2015). On Using Model Populations to Determine Mechanical Properties of Skeletal Muscle. Application to Concentric Contraction Simulation. Ann. Biomed. Eng. 43, 2444–2455. 10.1007/s10439-015-1279-6 25691399

[B182] Sivaraj K. K. Jeong H.-W. Dharmalingam B. Zeuschner D. Adams S. Potente M. (2021). Regional Specialization and Fate Specification of Bone Stromal Cells in Skeletal Development. Cel Rep. 36, 109352. 10.1016/j.celrep.2021.109352 PMC8293626 34260921

[B183] Soltan M. Rohrer M. D. Prasad H. S. (2012). Monocytes: Super Cells for Bone Regeneration. Implant Dent. 21, 13–20. 10.1097/ID.0b013e31823fcf85 22214990

[B184] Sparks D. S. Saifzadeh S. Savi F. M. Dlaska C. E. Berner A. Henkel J. (2020). A Preclinical Large-Animal Model for the Assessment of Critical-Size Load-Bearing Bone Defect Reconstruction. Nat. Protoc. 15, 877–924. 10.1038/s41596-019-0271-2 32060491

[B185] Stark R. Grzelak M. Hadfield J. (2019). RNA Sequencing: the Teenage Years. Nat. Rev. Genet. 20, 631–656. 10.1038/s41576-019-0150-2 31341269

[B186] Stéphanou A. Volpert V. (2016). Hybrid Modelling in Biology: a Classification Review. Math. Model. Nat. Phenom. 11, 37–48. 10.1051/mmnp/201611103

[B187] Steeve K. T. Marc P. Sandrine T. Dominique H. Yannick F. (2004). IL-6, RANKL, TNF-alpha/IL-1: Interrelations in Bone Resorption Pathophysiology. Cytokine Growth Factor. Rev. 15, 49–60. 10.1016/j.cytogfr.2003.10.005 14746813

[B188] Steiner M. Claes L. Ignatius A. Niemeyer F. Simon U. Wehner T. (2013). Prediction of Fracture Healing under Axial Loading, Shear Loading and Bending Is Possible Using Distortional and Dilatational Strains as Determining Mechanical Stimuli. J. R. Soc. Interf. 10, 20130389. 10.1098/rsif.2013.0389 PMC3730685 23825112

[B189] Stewart S. K. (2019). Fracture non-union: A Review of Clinical Challenges and Future Research Needs. Malays. Orthop. J. 13, 1–10. 10.5704/MOJ.1907.001 PMC6702984 31467644

[B190] Stoecklein V. M. Osuka A. Lederer J. A. (2012). Trauma Equals Danger–Damage Control by the Immune System. J. Leukoc. Biol. 92, 539–551. 10.1189/jlb.0212072 22654121 PMC3427603

[B191] Sun X. Su J. Bao J. Peng T. Zhang L. Zhang Y. (2012). Cytokine Combination Therapy Prediction for Bone Remodeling in Tissue Engineering Based on the Intracellular Signaling Pathway. Biomaterials 33, 8265–8276. 10.1016/j.biomaterials.2012.07.041 22910219 PMC3444627

[B192] Toben D. Schroeder I. El Khassawna T. Mehta M. Hoffmann J. E. Frisch J. T. (2011). Fracture Healing Is Accelerated in the Absence of the Adaptive Immune System. J. Bone Mineral Res. 26, 113–124. 10.1002/jbmr.185 20641004

[B193] Tourolle né Betts D. C. Wehrle E. Paul G. R. Kuhn G. A. Christen P. Hofmann S. (2020). The Association between Mineralised Tissue Formation and the Mechanical Local *In Vivo* Environment: Time-Lapsed Quantification of a Mouse Defect Healing Model. Sci. Rep. 10, 1100. 10.1038/s41598-020-57461-5 31980656 PMC6981157

[B194] Trejo I. Kojouharov H. Chen-Charpentier B. (2019). Modeling the Macrophage-Mediated Inflammation Involved in the Bone Fracture Healing Process. Math. Comput. Appl. 24, 12. 10.3390/mca24010012

[B195] Tsiridis E. Upadhyay N. Giannoudis P. (2007). Molecular Aspects of Fracture Healing: Which Are the Important Molecules? Injury 38, S11–S25. 10.1016/j.injury.2007.02.006 17383481

[B196] Tsuchiya S. Yamabe M. Yamaguchi Y. Kobayashi Y. Konno T. Tada K. (1980). Establishment and Characterization of a Human Acute Monocytic Leukemia Cell Line (THP-1). Int. J. Cancer 26, 171–176. 10.1002/ijc.2910260208 6970727

[B197] ASME (2018). Assessing Credibility of Computational Modeling through Verification and Validation: Application to Medical Devices New York, NY: The American Society of Mechanical Engineers.

[B198] Vaeyens M.-M. Jorge-Peñas A. Barrasa-Fano J. Steuwe C. Heck T. Carmeliet P. (2020). Matrix Deformations Around Angiogenic Sprouts Correlate to Sprout Dynamics and Suggest Pulling Activity. Angiogenesis 23, 315–324. 10.1007/s10456-020-09708-y 31997048

[B199] Van Dyke Parunak H. Savit R. Riolo R. L. (1998). “Agent-based Modeling vs. Equation-Based Modeling: A Case Study and Users’ Guide,” in Multi-Agent Systems and Agent-Based Simulation. Editors Sichman J. S. Conte R. Gilbert N. (Berlin, Heidelberg: Springer Berlin Heidelberg), 10–25. 10.1007/10692956_2

[B200] Vats D. Mukundan L. Odegaard J. I. Zhang L. Smith K. L. Morel C. R. (2006). Oxidative Metabolism and PGC-1*β* Attenuate Macrophage-Mediated Inflammation. Cel Metab. 4, 13–24. 10.1016/j.cmet.2006.05.011 PMC1904486 16814729

[B201] Vetter A. Witt F. Sander O. Duda G. N. Weinkamer R. (2012). The Spatio-Temporal Arrangement of Different Tissues during Bone Healing as a Result of Simple Mechanobiological Rules. Biomech. Model. Mechanobiol. 11, 147–160. 10.1007/s10237-011-0299-x 21431883

[B202] Viceconti M. Henney A. Morley-Fletcher E. (2016). In Silico clinical Trials: How Computer Simulation Will Transform the Biomedical Industry. Int. J. Clin. Trials 3, 37. 10.18203/2349-3259.ijct20161408

[B203] Virgilio K. M. Martin K. S. Peirce S. M. Blemker S. S. (2015). Multiscale Models of Skeletal Muscle Reveal the Complex Effects of Muscular Dystrophy on Tissue Mechanics and Damage Susceptibility. Interf. Focus 5, 20140080. 10.1098/rsfs.2014.0080 PMC4342948 25844152

[B204] Vodovotz Y. Chow C. C. Bartels J. Lagoa C. Prince J. M. Levy R. M. (2006). In Silico models of Acute Inflammation in Animals. Shock 26, 235–244. 10.1097/01.shk.0000225413.13866.fo 16912648

[B205] Wagar L. E. DiFazio R. M. Davis M. M. (2018). Advanced Model Systems and Tools for Basic and Translational Human Immunology. Genome Med. 10, 73. 10.1186/s13073-018-0584-8 30266097 PMC6162943

[B206] Wang M. Yang N. (2018). Three-dimensional Computational Model Simulating the Fracture Healing Process with Both Biphasic Poroelastic Finite Element Analysis and Fuzzy Logic Control. Sci. Rep. 8, 1–13. 10.1038/s41598-018-25229-7 29712979 PMC5928059

[B207] Ward P. A. Lentsch A. B. (1999). The Acute Inflammatory Response and its Regulation. Arch. Surg. 134, 666–669. 10.1001/archsurg.134.6.666 10367878

[B208] Warrender C. Forrest S. Koster F. (2006). Modeling Intercellular Interactions in Early Mycobacterium Infection. Bull. Math. Biol. 68, 2233–2261. 10.1007/s11538-006-9103-y 17086496

[B209] Wehrle E. Tourolle né Betts D. C. Kuhn G. A. Scheuren A. C. Hofmann S. Müller R. (2019). Evaluation of Longitudinal Time-Lapsed *In Vivo* Micro-CT for Monitoring Fracture Healing in Mouse Femur Defect Models. Sci. Rep. 9, 17445. 10.1038/s41598-019-53822-x 31768003 PMC6877534

[B210] Wendelsdorf K. V. Alam M. Bassaganya-Riera J. Bisset K. Eubank S. Hontecillas R. (2012). Enteric Immunity Simulator: A Tool for In Silico Study of Gastroenteric Infections. IEEE Trans. Nanobiosci. 11, 273–288. 10.1109/TNB.2012.2211891 PMC3715318 22987134

[B211] Wendler S. Schlundt C. Bucher C. H. Birkigt J. Schipp C. J. Volk H.-D. (2019). Immune Modulation to Enhance Bone Healing—A New Concept to Induce Bone Using Prostacyclin to Locally Modulate Immunity. Front. Immunol. 10, 713. 10.3389/fimmu.2019.00713 31024548 PMC6459956

[B212] Westman J. Grinstein S. Marques P. E. (2020). Phagocytosis of Necrotic Debris at Sites of Injury and Inflammation. Front. Immunol. 10, 3030. 10.3389/fimmu.2019.03030 31998312 PMC6962235

[B213] Wilkinson D. J. (2009). Stochastic Modelling for Quantitative Description of Heterogeneous Biological Systems. Nat. Rev. Genet. 10, 122–133. 10.1038/nrg2509 19139763

[B214] Yates A. Chan C. C. Callard R. E. George A. J. Stark J. (2001). An Approach to Modelling in Immunology. Brief. Bioinf. 2, 245–257. 10.1093/bib/2.3.245 11589585

[B215] Zahedmanesh H. Lally C. (2012). A Multiscale Mechanobiological Modelling Framework Using Agent-Based Models and Finite Element Analysis: Application to Vascular Tissue Engineering. Biomech. Model. Mechanobiol. 11, 363–377. 10.1007/s10237-011-0316-0 21626394

[B216] Zhang T. Yao Y. (2019). Effects of Inflammatory Cytokines on Bone/cartilage Repair. J. Cell Biochem. 120, 6841–6850. 10.1002/jcb.27953 30335899

[B217] Zhang Y. Böse T. Unger R. E. Jansen J. A. Kirkpatrick C. J. van den Beucken J. J. J. P. (2017). Macrophage Type Modulates Osteogenic Differentiation of Adipose Tissue MSCs. Cel Tissue Res. 369, 273–286. 10.1007/s00441-017-2598-8 PMC5552848 28361303

[B218] Zhang B. Korolj A. Lai B. F. L. Radisic M. (2018). Advances in Organ-On-A-Chip Engineering. Nat. Rev. Mater. 3, 257–278. 10.1038/s41578-018-0034-7

[B219] Zienkiewicz O. C. Taylor R. L. Nithiarasu P. Zhu J. (1977). The Finite Element Method, 3 London, UK: McGraw-Hill.

[B220] Zura R. Xiong Z. Einhorn T. Watson J. T. Ostrum R. F. Prayson M. J. (2016). Epidemiology of Fracture Nonunion in 18 Human Bones. JAMA Surg. 151, 1–12. 10.1001/jamasurg.2016.2775 27603155

[B221] Zysset P. K. Dall’Ara E. Varga P. Pahr D. H. (2013). Finite Element Analysis for Prediction of Bone Strength. BoneKEy Rep. 2, 1–9. 10.1038/bonekey.2013.120 PMC3765052 24422106

